# Viral time capsule: a global photo-elicitation study of child and adolescent mental health professionals during COVID-19

**DOI:** 10.1186/s13034-021-00359-5

**Published:** 2021-02-02

**Authors:** Olivia D. Herrington, Ashley Clayton, Laelia Benoit, Cecil Prins-Aardema, Madeline DiGiovanni, Indigo Weller, Andrés Martin

**Affiliations:** 1 Yale School of Medicine, New Haven, CT USA; 2 Department of Psychiatry, Yale School of Medicine, New Haven, CT USA; 3 Public Health and Sociology, Inserm (Institut National de La Santé et de La Recherche Médicale) and CESP (Centre de recherche en Epidémiologie et Santé des Populations), Paris, France; 4 GGZ Drenthe (Geestelijke Gezondheids Zorg: Mental Health Care), Beilen, The Netherlands; 5 Center for Educational Development and Research in Health Sciences (CEDAR), University Medical Center Groningen, Groningen, The Netherlands; 6 Bioethics Program, Harvard University, Cambridge, MA USA; 7 Child Study Center, Yale School of Medicine, 230 South Frontage Road, New Haven, CT 06520-7900 USA

**Keywords:** Photo-elicitation, Professional identity, Mental health professionals, COVID-19 pandemic, Qualitative methods, Thematic analysis, Rich picture analysis

## Abstract

**Objective:**

To examine, through photo-elicitation, the personal and professional impact of the COVID-19 pandemic on mental health professionals working with children and adolescents around the globe.

**Methods:**

We invited the submission of images collected about the pandemic between May and August 2020. We encouraged participants to yoke personal reflections or voice memos to their images. Using snowball sampling, we began with two invitations, including one to the graduates of a mentorship program continuously hosted since 2004 by the International Association of Child and Adolescent Psychiatry and Allied Professions (IACAPAP). We analyzed de-identified images and anonymized transcripts through iterative coding using thematic analysis informed by rich picture analysis and aided by NVivo software.

**Results:**

We collected submissions from child and adolescent mental health professionals (n = 134) working in 54 countries spread across the five continents. We identified four overarching domains with component themes that revealed both the commonality and the uniqueness of the pandemic experience around the globe: (1) *Place* (adjusting to emptiness and stillness; shifting timeframes; blending of spaces); (2) *Person* (disruption to life rhythms; emotional toll; positives of the pandemic); (3) *Profession* (changing practices; outreach efforts; guild pride—and guilt); and (4) *Purpose* (from pandemic to syndemic; from lamenting to embracing; planning toward a better tomorrow).

**Conclusions:**

Photo-elicitation provided a disarming and efficient means to learn about individual, regional, and global similarities and differences regarding the professionals charged with addressing the mental health needs of children and adolescents around the globe. These findings may help inform practice changes in post-pandemic times.

*For a better world to emerge after this pandemic, we must embrace and nourish the feelings of humility and solidarity engendered by the current moment.*

- Orhan Pamuk [1]

*What if 2020 isn’t cancelled? / What if 2020 is the year we’ve been waiting for? / A year so uncomfortable, so painful, so scary, so raw – / That it finally* forces *us to grow. //*

*2020 isn’t cancelled, but rather / the most important year of them all.*

- Leslie Dwight [2]

## Background

Societal adaptations to the spread of the Sars-CoV-2 virus that started in late 2019 have fundamentally altered daily life across the globe. In part because of these alterations, the COVID-19 pandemic has had a profound effect on the prevalence and natural history of mental health conditions. Much of this influence is rooted in social isolation attributable to lockdown, shelter-in-place, and quarantine practices [3, 4]. Such isolation has been known to increase stress and worsen mental health in situations as varied as epidemics and terrorist threats [5].

The psychological consequences of the pandemic have already had a significant effect on the lives and experiences of children and families [6]. Overwhelmed by expectations to simultaneously work from home and care for their children full time, or to fulfill obligations placed on them as essential workers, parents have struggled to meet their children’s emotional needs, as well as their own [7]. Worse still, many parents have lost their jobs entirely, leaving their families vulnerable to the adverse consequences of socioeconomic adversity [4]. The increased social and emotional pressures of this unheralded time have already elevated rates of abuse, neglect, and interpersonal violence [7, 8].

In addition to an increase in risk factors, there has also been a reduction in protective factors. Access to usual outlets and coping mechanisms like after-school activities, playgrounds, and time with friends and extended family has been dramatically restricted. School closures have removed a source of material support, such as free and reduced-price meals for children from low-income families, as well as of psychosocial support. School closures have also reduced the likelihood that abuse and neglect are recognized and intervened upon, as children now rarely encounter adults outside the home [4, 9]. Indeed, even seeking help for abuse and interpersonal violence over the phone has become challenging in many households, where crowding and tight quarters have infringed upon access to safe and confidential spaces for conversation [8].

Mental health professionals routinely work with children and adolescents in their home and school environments. The “ecosystem” in which a child lives is one of the main targets for mental health interventions. Professionals work to change and improve such environments and empower children and families where they live. The pandemic has limited, when not altogether deprived, mental health professionals of one of their best working tools: the everyday settings of children’s lives.

Healthcare professionals are not immune to the stresses and anxieties of life under pandemic conditions either. Clinicians and other medical personnel on the frontlines of disease outbreaks like MERS, Ebola, and Sars-CoV-1 experienced a significant increase in symptoms of PTSD, anxiety, and other psychiatric illnesses [5]. The COVID-19 pandemic has been no exception to this pattern. A study in China found that most healthcare workers in the midst of the pandemic experienced psychological distress, with nearly half endorsing significant symptoms of anxiety and depression [10]. Even at a non-pathological level, there has been widespread anxiety around not having access to sufficient personal protective equipment (PPE), being a possible vector of disease to one’s family, and performing unfamiliar roles within the healthcare system [11].

In this study, we use photo-elicitation and qualitative methods to examine the personal and professional impact of the COVID-19 pandemic on mental health professionals working with children and adolescents across the globe.

## Methods

### Design

We based our qualitative study on photo-elicitation methodology [12], specifically on the approach variously termed “reflexive photography,” “auto-driven photo-elicitation,” or “photo-voice,” [13, 14] in which respondents provide meaningful images from their unique points of view and for which they provide related reflections. Photo-elicitation has been routinely used as a methodology in visual anthropology and sociology and has made inroads into healthcare, including in child psychology [15]. Photo-elicitation was first described as a variation on open-ended interviewing: a nondirective method that fosters the collaboration between researcher and respondent [16]. Photographs can convey content that words can only approximate, eliciting unforeseen meanings and interpretation [17], making for a polysemic process of multilayered meanings for a given stimulus.

### Participants and ethics approval

We invited child and adolescent mental health professionals to submit images representative of their experiences during the COVID-19 pandemic. We encouraged participants to yoke personal reflections or voice memos to their images and placed no limit on the number of images or reflections they could submit.

We approached individuals through two separate invitations: (1) Locally, to all members of the Yale Child Study Center community, encouraging submission of entries toward a grand rounds presentation entitled “Selfies Gone Viral,” which was held virtually on May 12, 2020; and (2) Internationally, to graduates of the Donald J. Cohen mentorship program (DJCP), which has been continuously hosted since 2004 by the International Association of Child and Adolescent Psychiatry and Allied Professions (IACAPAP) [18–20]. Snowballing from these two outreach efforts yielded our final study sample.

We obtained ethics approval from the Yale University Institutional Review Board (Protocol #2000028102). Our study was deemed exempt under 45CFR46.104 (2)(ii) and did not require the written informed consent of its participants. We provide results without inclusion of any potentially identifying personal information. We obtained separate written releases for those images included in Additional file 2: Appendix S2, which contain potentially identifiable visual information.

### Data collection

We used the following language in our e-mail invitation, which was open between May and August 2020:

We invite you to share with us images that represent your work during the pandemic. Specifically, we are looking for images that encapsulate the interface between your inner/personal and outer/professional lives. Our specific ask is to consider sharing two things: an image (or more), and a written or voice memo reflection of your thoughts related to the image(s). The reflection does not need to be polished—indeed, we encourage you to be spontaneous and not self-censored. We enclose examples from our respective work in order to give you an idea of what we have in mind.

This entreaty led to two sources of submission types: (1) Images, comprising pictures taken with digital cameras or cell phones, including: “selfies,” screenshots, cartoons, drawings, paintings, memes, and video recordings; and/or (2) Written reflections provided as emails, voice memos, longer documents, and text or WhatsApp messages. Deidentified transcripts formed a third data source from: (3a) a recording of the grand rounds held in May, and (3b) two 90-min “photojam” sessions held in June and July, during which the authors reflected as a group on a collated selection of the images received to date.

We uploaded all data into a relational database (NVivo version 12; QSR International, Melbourne, Australia) for the purposes of file organization and software-supported qualitative analysis. We conceptualized our dataset as a virtual time capsule for the experiences of a unique group of individuals (child and adolescent mental health professionals) during a circumscribed time of historical relevance (4 months early on in the COVID-19 pandemic). Time-capsule methodology is commonplace in the fields of archaeology, anthropology, history, and ethnography [e.g. 21], and has been valuable in areas as diverse as the law [22] and geriatric medicine [23].

### Data analysis

We analyzed the transcripts using thematic analysis [24, 25], which provides theoretical freedom and flexibility to identify commonalities, and in which writing and analyzing data occur recursively alongside one another. Inductive thematic analysis within a constructivist framework includes a rich and detailed account of the data and welcomes and indeed encourages attention to the investigators’ reflexivity [26]. Our approach was *inductive* insofar as it built from data up to theory, rather than moving from pre-existing theory down into buttressing data, as would have been the case in a deductive approach. We incorporated additional analytic strategies commonly used in: (a) constructivist grounded theory (CGT) [27], including purposive sampling, iteration, co-construction, and memo writing to explore the relation between complex images and their associated reflections, including the investigators’ personal and subjective views; and (b) “rich picture” scholarship, in which visual methods help explore complex phenomena and understand how people experience and give meaning to such complexity [28, 29].

Three authors (OH, AC, AM) worked independently to identify and compare codes before removing redundancies, sharing them with the other investigators for further refinement, and finalizing them into a joint codebook of overarching themes that reached *theoretical sufficiency* [30], the point at which additional data do  not contribute further to the development of a given theme, or to the creation of a new one.

Each key theme was supported by multiple quotes. In keeping with the tenets of participatory research [31], we value the perspective of all participants, and invited them to review and comment on our final codes, overarching conclusions, and manuscript draft. Five of the contributors are co-authors of this report.

## Results

### Sample description

**Fig. 1.**
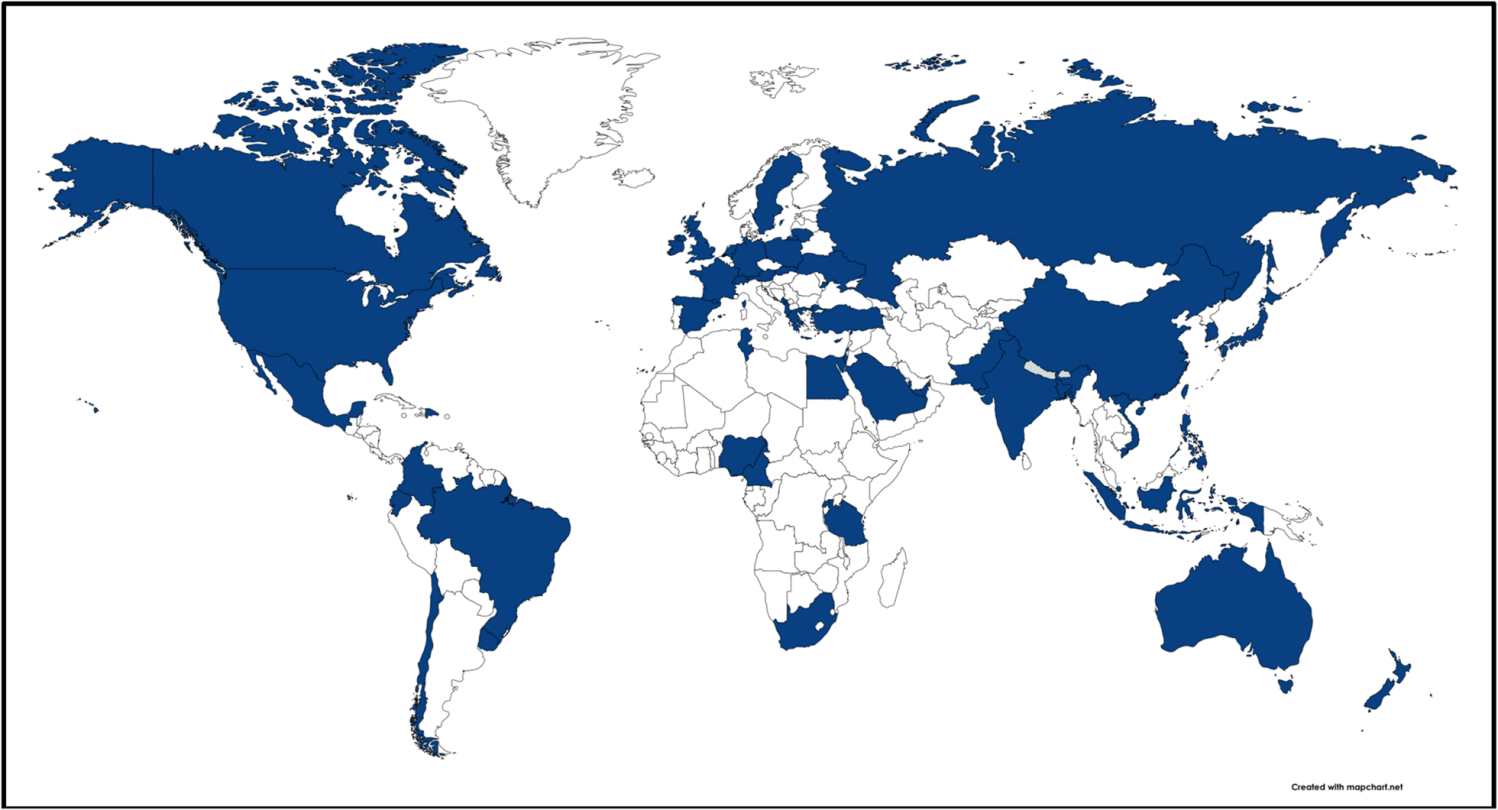
Countries with at least one photo-elicitation contributor to the study. World map created with mapchart.net based on 134 photo-elicitation entries spanning 54 countries, as tabulated in Additional file 1: Appendix S1.

We obtained submissions from individual child and adolescent mental health professionals (n = 134) working in 54 countries spread across the inhabited continents. Figure 1 provides a visual representation of the countries with at least one photo-elicitation contributor to the study. Additional file 1: Appendix S1 tabulates those countries, organized by continent and World Bank classification [32].

Initial submissions derived from the grand rounds presentation at Yale (n = 22, representing 16% of the sample) and from the 20 DJCP leaders we approached (two for each of ten cohorts, 14 of whom responded, for 10% of the sample). The remaining submissions (n = 98, representing 73% of the sample) snowballed from the DJCP invitation by the investigators and from personalized reminders by cohort leaders. Since its inception sixteen years earlier, the DJCP ha enrolled a total of 432 participants, 286 (66%) of whom had active email addresses accessible to us. This translated into a DJCP response rate of 112/286 (39%) among those alumni we were able to contact.

Participants submitted images (n = 22 [16%]), written reflections (9 [7%]), or a combination of the two (103 [77%]). The median number of images submitted was 2 [range, 1–24] among the 125 (93%) who submitted images. Overall, our database included a total of 278 images.

### Thematic analysis

Through iterative thematic analysis, we developed a four-part model along the alliterative domains of *Place, Person, Profession*, and *Purpose*. We go on to deconstruct each domain in the sections and tables that follow. We include image examples in support of these themes in Additional file 2: Appendix S2.

## Domain 1. Place (Table 1)

**Table 1:** Place: themes and sample quotes

Theme	Sample quotes *(source country)*
1.1 Adjusting to emptiness and stillness	The most striking thing about these walks, apart from the beauty of the view, was the lack of airplanes in the sky. Our airport is usually very busy and although it was nice to feel so calm, no aircraft made everything seem eerie, a bit like an apocalypse movie. *(Switzerland)* “What’s happening?”, “Where are all the patients?”, “How does it come to be so?”, “Why does the hospital look like a ghost town?”—many questions emerged. However, there was only one answer fitting all these concerns: the reason is COVID-19, the consequence is doctors without patients. *(Vietnam)*
1.2 Shifting timeframes	I had seldom, if ever, felt so disconnected and disoriented—not only from important people in my life but also from the rhythms that had been familiar and recognizable to me as my life. *(USA)*
1.3 Blending of spaces: inside and out, personal and professional	Our provincial government had ordered all public parks, playgrounds, and trails closed. Officials had taped off entrances to facilities, basketball hoops and, in this case, an urban playground…I was focused on the abundance of tape, and the rattling metaphor that it conveyed about what this pandemic would mean to the concepts of childhood, parenting, and fun. *(Canada)* Maybe it was OK to show that I was in my apartment, that behind me was my messy and unruly kitchen. I don't know. I felt maybe too exposed…I saw this kind of tension between being open, accepting to share a little bit more than usual with my patients, while keeping a sense of privacy. *(France)*

### 1.1 Adjusting to emptiness and stillness

Many participants submitted photos of landscapes that would normally be bustling with signs of human life, such as parks, bridges, and busy highway exchanges, now empty. Some participants described how their external topography had visually changed, as if the world, once seemingly occupied by people, now felt unfamiliar or unnerving:

We have been living in a dystopic reality for the past four months, since schools, universities and all non-essential businesses are closed. In the first days, I remember looking out of the window to a usually very busy avenue on Monday mornings: I could not see cars or persons for a few minutes, which was very disturbing in a city that never sleeps. *(Brazil).*

Words like “eerie,” “apocalyptic,” and “ghostly” were common companions to the unpopulated landscape images. Some wrote of a mixture of feeling both calm and unsettled at once, others noted how such bareness allowed them to better appreciate the beauty of the outside world, and yet others struggled with the privilege of bucolic reflection:

I decided to look out from my rooftop garden and it occurred to me that I might be living in an ivory tower that does not allow me to see what is happening on the ground every day, all the time. *(Singapore).*

In addition to barren landscapes, participants submitted photos of empty spaces, such as schools and outpatient clinics. Clinicians wrote about the impact of the loss of usual, in-person services on both their patients and themselves:

Patients disappeared from all clinics, not only from ours. Community services rapidly adapted to the new reality, but also few patients appeared. By mid-June, patients started to come back, worsened. *(Brazil).*

No patients, no appointments for a normal working day. We call this: “a unique event in a unique time.” *(Vietnam).*

Some wrote of the emotional challenge of adjusting to empty spaces and life with fewer human-to-human interactions. Even visual vocabularies needed readjusting, as noted by a clinician–painter who coped through crafting a series of watercolors near the start of the pandemic:

When every city in the world became a Hopper painting, with no people and no life, it was hard for me to paint empty streets any longer. *(Spain).*

Participants wrote not only of the visual absence of others but also of the “deafening silence” that accompanied this absence:

Avoiding the fellow humans responsible for this glorious noise and color has been a bizarre and dreadful adjustment. But I have adjusted. *(USA).*

Others spoke of “unseasonably cold air” and other embodied experiences of emptiness during the pandemic. In some instances, there was a sense of foreboding and impending danger, as the scale of the pandemic began to take hold:

This kind of visceral, full body experience, when you know something is not right. I grew up in an area of the country where tornados were not unusual. Everything gets very still. All of a sudden, the sky is this weird shade of green. Before you describe it, you feel it in your stomach. *(USA).*

### 1.2 Shifting timeframes

Reflecting back to other times helped make sense of such an unprecedented situation. Confronting the pervasive emptiness head on, one child psychiatrist explored abandoned psychiatric facilities in her free time:

It is hard not to see an abandoned hospital as an 8,000-square-foot *memento mori*. I am seeking out a series of liminal spaces during a societal period of liminality…Timelines have collapsed. *(USA).*

As days and weekends merged into “Blursday” and the social rhythms of life became scarce, the sense of time became relative, malleable. Recalling past events or thinking ahead to better times became a commonplace form of mental time travel. Past events, including war and other catastrophes, served as a framework through which to come to terms with mounting loss and devastation:

Why is it that I think back to that terrible day so many years ago? It may be that as we live through this pandemic, this tragedy of a very different kind, one that we cannot see, one that is not circumscribed and finite, and one whose repercussions are likely to stretch for months and years, if not indefinitely, I think back to those terrible few hours. Where will we be as many years from now? What will be the repercussions of the invisible scourge we are living through? I do not know, but on this day, with blue skies, fair weather and the ability to go out and celebrate life on my bike, I think not of the tragedy that we lived through back then, or of the one we are living through these days, but of the gifts of endurance, of perseverance, of life and love. Final score for the day: life one, death zero. *(USA).*

A yearning to be at another time undergirded the direct confrontation of the first quote, the reliving of past traumatic experiences that of the second. Both instances exemplify the mental escapism through fantasy, exercise, or art to which so many resorted during the chaotic early months of the pandemic.

### 1.3 Blending of spaces: inside and out, personal and professional

Images of outdoor spaces in which children typically play, now closed and out of reach, elicited sadness about young children and relatives who “stayed inside for months… denied all play…not allowed out” *(Spain).* The potential social and psychological toll of life under pandemic conditions on children’s social and emotional development, especially the younger ones, was at the forefront of many reflections: what it “would all mean to the concepts of childhood, parenting, and fun” *(Nigeria).*

The “outside” spaces of the world at large resonated through travel restrictions and the severity and impact to move freely around the world. Reflecting on other dark times,

We who were born here still remember very well an iron curtain that did not allow us to travel abroad. We all have an inborn fear that this could happen again, but that the borders could be closed from the outside was simply unimaginable. *(Russia).*

Living outside of their home countries, some participants submitted images representing the challenge of deciding whether to return home, if even possible, or shelter in place in their current locations without a clear timeline for when international borders would reopen. With such restrictions on movement and space usage, sabotage of public health recommendations against viral spread stuck out:

That very insular community in large part threw caution to the wind and maintained prayer and learning centers open, crowded tight as always. The percentage of COVID-19 positive cases in that community were staggering. Literally overnight, we had to open two full corona wards, including a large corona ICU unit. *(Israel).*

Such cavalier, self-serving, disbelieving or careless behaviors placed others at risk, and led to reflections on the jarring exposure to human indifference.

The clear delineation between personal and professional spaces that many had taken for granted was overtaken by a merging of the two. For professionals who are regularly cognizant of and attuned to boundaries and demarcations, this proved to be disorienting, more so given that outpatient child psychiatry—unlike, say surgery—can in fact be practiced from another kind of bedside: the practitioner’s bedside. Images depicted the melding of personal and professional spaces, and reflections addressed both the opportunities and the risks that telehealth visits posed to the therapeutic frame. Figuring out what aspects and just how much personal life to showcase and share with patients was a commonly addressed conundrum: “my makeshift office on the kitchen table is a good representation of the artificial splitting I’ve had to impose on myself.” *(Turkey).*

Clinician-parents appreciated more time with their children at home but wrote of the stress and challenges of practicing telehealth, “including how to manage to get through a 30-min patient meeting without having my three-year-old daughter find her way to the office wanting to show me her favorite toy!” *(Sweden)* A related, and common, concern was the psychological impact of delivering clinical care in the physical space of one’s home: “How do we leave our work at those doors, right in the space where we did our sessions? How do we go from session after session of consoling families and helping them manage their crises only to turn around and console our own families and ourselves?” *(Australia)* The separation between on and off modes also seemed to dissipate through the

various struggles that have come about now that all of my roles actively need me. Normally if work is demanding, my family life is stable, etc. But now, every single identity/role needs something from me. *(USA)*

## Domain 2: Person (Table 2)

**Table 2:** Person: themes and sample quotes

Theme	Sample quotes *(source country)*
2.1 Disruption to life rhythms, family impact	Children can’t go out and enjoy the summer, can’t see their grandparents, some are dead, some are too afraid of getting the virus, they can’t see their teachers or school friends. Driving through the city, our children asked, “Wait, STOP, our school is there!, can you stop a moment so we can see it?” *(Spain)* The visitor restrictions have been among the most challenging pieces of this puzzle. Knowing that family is so important and necessary for the children, for them to not feel abandoned, for them to know that they're supported, has been one of my main goals throughout this pandemic. It's very difficult to explain to them the necessity of social distancing, why we're wearing masks, why they are wearing masks. *(USA)*
2.2 Emotional toll, loss and death	In a matter of days we’ve gone through all the stages of grief and loss, from initial denial and isolation (“it will not happen here; it is a China and Italy problem, our health system is the best in the world”), to anger (“who’s fault is it?…human’s best friend is not the dog, it’s the scapegoat”). Then bargaining (“it is bad only if you are very old or already ill”) and then you see young healthy people dying, or having amputations due to thrombosis, or pulmonary embolisms. Then depression (“my God, it IS here, and there is nothing or little I can do”), and finally acceptance…I am not quite there yet. This reality is difficult to accept. *(Spain)* This situation has in some subtle way robbed us from the absolutism of daily life. The things we took for granted are now no longer reliable. The belief that we had in the world: that optimism, that naiveté, have been taken away. And we are terrified. Death was a distant and abstract entity. The COVID pandemic in no time has collectively given us a reality check and here we are, perplexed…Truth came very abruptly: the truth of our mortality, our vulnerability, our feeble existence in the face of this adversity. It reminds me of something I read years ago: “Just when I have all the right cards, everyone else stars playing chess”. We thought we had figured it all out. *(Pakistan)*
2.3 Positives of the pandemic	Family, family, family. Every cloud has a silver lining. *(Brazil)* It may be self-indulgent to say so, but when again will I have all this time to make productive use of? It Is remarkable how much time I’ve reclaimed just by not having to run around from one physical location to another. *(Israel)*

### 2.1 Disruption to life rhythms, family impact

Disruptions to life rhythms and routines were most often described in negative terms: “Our routines are changed, the children’s routines are changed, and it just doesn’t always feel so good” *(Ireland).* Another wrote of the isolation experienced as a result of the interruption to regular practices “as I tried to find a new sense of routine and ritual in the ever-changing landscape of the city” *(China).*

Some submissions memorialized disrupted rituals and rites of passage, such as a screenshot of a Ph.D graduation whose recipient wrote about how “there were no congratulations, no celebration, just a click on a keyboard, and it was gone” but hoped that the Ph.D celebrations were “perhaps opportunities deferred rather than lost” *(Nigeria).* One training director wrote of the grief experienced from “the fact that my fellows will be leaving, and we can’t give them a proper send-off.” *(USA).*

Disruptions affected a range of lifecycle milestones, just as they emphasized the resilience brought to bear in order to endure the pandemic. Starting with childbirth and the reclaiming of comfort, warmth, and normalcy within the chaos,

We could never have anticipated that I would be delivering our first child in the midst of a pandemic. We had planned meticulously: our parents and siblings were going to visit in turns to help us with the baby. And here we were, discharged less than 24 h after birth (to minimize risk of infection) holding our child tightly, and grateful to be together in our isolation. As we gradually sank into the deep and mellow gratitude of our new normal, I could not shake off the feeling that many people had been without any support during childbirth. Still others were dying alone in hospitals from the virus *(USA).*

Cultural mores were affected, as in the suspension of a traditional greeting, which led to the re-appropriation of a variation first used in 1918:

Culturally our practice of *hongi*, the sacred sharing of each other’s breath through touching noses, was not allowed. I was in grief at this sudden loss. One of our leaders came up with a greeting that had first been used in the time of the Spanish flu. This was resurrected as *hā mamao*, literally, breath from a distance. This became our new salutation. *(New Zealand).*

Some were used to living with a higher threshold of unpredictability than others, yet even they recognized the profound impact the pandemic would have on the world. One participant wrote, “Growing up in my intensely diverse and vibrant metropolis, one learns to live with a certain amount of uncertainty and unpredictability…but when I first read about COVID, I knew the world would not be the same again.” *(Pakistan).*

The toll on family life was pronounced for many, particularly those with young children suddenly left without the structure and support provided by their schools. Experiencing the competing demands of parenting and doctoring became very real during clinical interactions:

Families are left breathless by the new demands of our shelter-in-place life: trying to feed, homeschool, entertain and soothe all of their children, while not missing a beat of their employer’s ceaseless drum…I find myself saying again and again: “You’re right, there *aren’t* enough hours in the day. There simply are not.” There aren’t in my home either: we try to keep up with the flurry of emails from our child’s school about virtual assignments and school portals, each, of course, with its own login system and password, overtaxing our neurons to the point where they begin to fray. *(USA).*

By not permitting, or severely curtailing, family visits, emergency department or inpatient settings posed additional challenges. These could lead to wrenching decisions about the optimal course of care:

I think of the brave parents who have to make the difficult decision of sending their children to an inpatient setting for safety in the midst of this. During my last on-call duty, the mother of a COVID-positive adolescent expressed how helpless she felt between getting the care her child so desperately needed and leaving him in isolation as she took care of the rest of her family. These are the difficult situations many parents and guardians have had to face *(USA).*

### 2.2 Emotional toll, loss, and death

Language and word choices reflected ways in which participants took stock of the emotional toll of the pandemic and coped with it:

“Unprecedented,” “uncertain,” “once-in-a-lifetime,” “unparalleled” are some of the words being used to describe this global event. It is hard to write a coherent reflection about something that continues to elicit a cacophony of reactions. *(USA).*

Our language becomes casually, but consistently the language of war: “First line,” “losses,” “additions,” “reserves,” “casualties.” How will this “battle” end? Unfortunately, everything seems to indicate that it will be a long positional “engagement.” *(Poland).*

Allusions to war, when not outright recollections of it, spoke to the deep emotional impact of months under *lockdown*, a term that itself “felt wrong, sounding military, with an air of finality.” *(New Zealand)* One participant wrote how

the coronavirus pandemic brought back personal and difficult memories of growing up at a time of civil war, when we were sequestered in our homes for extended period of time with rationing of food supplies and outdoor curfews. *(Cyprus).*

As weeks turned into months with an uncertain future, greater familiarity and ease with virtual platforms offered a way to reconnect and reach out to others. Supportive networks, from local and regional levels through to the international, opened up educational, networking, and outreach opportunities that had not been previously accomplished:

I talked to people I hadn’t spoken to before. There was an opening up of social interactions, a sense of license to converse, albeit at a distance. A drive to try to connect, to maintain some human bonds, to share the uncertainty and fear *(New Zealand).*

Coping through electronic connectivity occurred not only at the individual but also at the group level: robust attendance was reported for events that had no option but to switch to virtual platforms. As physical attendance became more constrained, emphasis shifted to mental presence and interpersonal connection. Without the gross inefficiencies inherent in traveling to and from meetings,

one of the paradoxes of this time was maintaining and even strengthening connection and community, given the massive reconfigurations of social space in which everyday gestures of care and concern had seemingly been disrupted. *(France).*

Grief was a pervasive theme across many submissions. Losses ranged from forgone routines and the hitherto taken-for-granted freedom of movement to a deeper sense of the affective and relational toll exerted by the long period of enforced social isolation:

Mark Epstein lays it out very beautifully in *The Trauma of Everyday Life* [33]. He talks about how this human attribute of relating to each other is assumed to be one’s birthright. If we look at the current pandemic situation, does it mean that COVID-19 has robbed us of our birthright? *(Pakistan).*

For those afflicted by the virus, the infection could concretize the loss of health—and potentially of life—in harrowing ways. Having enough medical knowledge to understand some of the unfolding events but not enough to address them made for a frightening lack of agency during the course of a personal illness. A physician recalled,

I spent more than a week at the hospital. My son had been hospitalized five days before me with an acute respiratory distress presentation. His situation was so frightening, and my desperation so profound, that my stress-weakened immune system could not fend off the virus. In the hospital, I faced a cytokine storm that could barely be slowed down by a combination of anti-IL6 medication, an antiviral, and convalescent plasma. After a few hours, the storm and my fever were over. I embraced the treatments, even as there was little evidence for their use. I also experienced corona hepatitis, a syndrome first described just a week before I had it. Due to the seriousness of my son's and my own condition, I had been in a dark mood and was just getting into a relatively reasonable state of mind, less fearful. However, news of the death of the physician with whom I had been admitted to the COVID floor reactivated my feelings and thoughts from the time of hospitalization—along with feeling guilty about having survived while others fell. My son and I have fully recovered. We now look for what we can do to stay human, to move forward and repurpose our lives and professions *(Turkey).*

The scale of mass deaths across the world was overwhelming and hard to reckon with:

So many people dead, so fast, that funeral homes, crematoria and undertakers can’t keep up. They had to store corpses in an ice-skating stadium known as “The Ice Palace.” A painfully ironic name for these poor people who died alone, most having never been to a palace before *(Spain).*

Having to attend virtually to the funerals and wakes of loved ones added to the disorienting and dreamlike quality of the times. For clinicians working in hospital settings or otherwise unable to fully isolate, the proximity to infection and death had an urgent immediacy, for which the pace of work precluded adequate emotional processing:

Some of us, myself included, experienced the grief of losing friends who passed away because of COVID-19. And that's a very hard thing to deal with, to bring that fear every day to work. But we simply had to keep moving on *(Brazil).*

### 2.3 Positives of the pandemic

Although the pandemic dramatically upended everyone’s lives, many submissions were of happy children, rich nature scenes, and bright colors. Caregivers wrote about the joy of spending more time with their family: “the amazing thing about having my kids back at home is that our house has been filled with music, round the clock” *(Canada).* Some participants shared images or wrote about the addition of a new pet to the family, the joy of watching their children reconnect to the things they love, or witnessing their children become helpers and engage in civic responsibility. One psychiatrist captured an image of his children building face shields to help ensure that frontline workers had PPE:

We may think that as child psychiatrists we know a whole lot about childhood, but it is children themselves who are our best teachers…reminding us of where ingenuity, breakthroughs and true hope will always stem from *(USA).*

Participants discussed finding new hobbies or rekindling old interests, from baking focaccia to gardening to cycling and spending time outdoors. A retired psychiatrist blessed with a large family captured the sentiment well (Table 3):

One of the positives of the lockdown is having more time on hand for activities and hobbies that are often sidelined. So I have been able to get on with painting the portraits of our eight grandchildren as young children, having started years ago with the eldest who is now 18 years and at University. I recently completed grandchild number 6, as shown in the picture, and am about to embark jointly on the last two – 8-year-old twins. I’m not sure what I will do if I run out of grandchildren before the pandemic ends *(South Africa).*

## Domain 3: Profession (Table 3)

**Table 3:** Profession: themes and sample quotes

Theme	Sample quotes *(source country)*
3.1 Changing practices	I was part a group of physicians from different specialties who volunteered to cover the newly established COVID-19 call center to handle calls from patients, families, other physicians and healthcare staff working in remote areas. This was a brand new experience for all this group, most of whom had never handled this type of patient before but now had to deal with a flood of calls, to make quick decisions, to handle cases as best we could while working in crisis mode. *(UAE)* And it has become more evident than ever to me that, the precious moments that you get to spend in a patient's room, they mean more than ever before. I know that we're supposed to be in the room of a COVID-positive patient as little as possible. And we're totally geared up, we have our masks, face shields, and gowns –and our patients can't really see our faces. But, it's hopefully through our eyes and the inflection of our voice that we are bringing hope and encouragement and trying to get these patients home as quickly as possible, as safely as possible. *(Redeployed psychiatric nurse, USA)* I called a family from far away, abroad. Mom and the adolescent girl came to the camera, and the grandparents also sat down in front of the computer to "see" the psychiatric visit—forget confidentiality and HIPAA violations: this is war. Probably they had nothing better to do that to see a live child psychiatric evaluation unfolding, no Netflix required. *(Uruguay)*
3.2 Outreach efforts	After the lifting of the lockdown I went out to distribute facemasks to traders at the market. The majority of them are uneducated and untrusting; they don’t see the need to buy or use facemasks. Most are of the opinion that since the government is enforcing it, they should have given out the facemasks freely to all citizens. *(Nigeria)*
3.3 Guild pride—and guilt	Despite a medical condition placing her at risk, [the nurse] didn’t bat an eye as she told me "I am here. This is my work. This is what I do. I will take extra and additional precautions in doing what I do, but I need to be here for the kids and it's a privilege to serve." And I sit in awe. In awe of what everybody is doing this day. I have fallen yet again back in love with medicine, with child psychiatry and with the wonderful, selfless people I get to work with each day. *(USA)* The stress we absorbed is starting to surface only now. The disconnect was difficult for me when hearing from some people how lockdown had been "a holiday". I found this tough to reconcile with my experiences of families struggling with intergenerational mental illness, substance abuse, violence, food insecurity, loss of work, loss of a future. The stark reminders of the ‘haves’ and ‘have-nots’ in our little corner of the world. *(New Zealand)*

### 3.1 Changing practices

The clinical practice of traditional outpatient child psychiatry changed rapidly. The shift was smoother for those already practicing telehealth, particularly in areas of the world with well-resourced governments and health systems able to support the virtual transition. The pandemic may have only accelerated inevitable trends: “we were quickly allowed to take care, from our respective cities, of patients anywhere in the country. For our large and sparsely populated nation with a shortage of child psychiatrists, telehealth will be the way of the future.” *(Australia)*.

For others, the experience was to become altogether different practitioners, to take on new and unfamiliar roles as they were redeployed, provided psychiatric services in new areas, or volunteered where they were most needed:

We had to remain psychiatrists (still and first of all), but also to become pulmonologists, reanimation-ologists, radiologists (“doc, I’m sure I swallowed it [the virus], could you please take a look?”), laboratory doctors, parents, and so much more. By the end of my shift in the hospital I hardly remembered what I wanted to say to this child in the morning *(Russia)*.

Disoriented professionals tried to find their bearings in suddenly unfamiliar territory. This was most palpable within hospital settings, where virtual meetings could not entirely supplant care:

For hours we analyze the paths we should take to and from patients infected with COVID-19 so that the paths of those who enter and leave patients do not intersect. We learn how to don (important) and how to doff (crucial) our PPE: barrier aprons, masks, goggles, helmets, protective scarves, shoe protectors. *(Poland)*

About 20% of our overall workforce across the hospital got sick, but slightly more than that got sick in our department –maybe from this sniffling, fever-spiking, runny-nosed kid, or perhaps more likely from their asymptomatic coevals– so we were constantly adjusting schedules, with new testing algorithms, new plans, new schedules, new PPE regulations *(USA).*

In the context of such highly restricted physical proximity, even of outright bans on physical contact, practices changed swiftly. Thinking of those infected with the virus, a psychiatric nurse redeployed to a COVID unit realized

how you're the patients’ only lifeline, the only person going into the room, the only person they get to physically see or touch – through a glove *(USA)*.

Whenever still possible, face-to-face care became mask-to-mask care. The introduction of PPE to psychiatry was unfamiliar and came with its own learning curve, a challenge to protect the therapeutic alliance given that

we rely so heavily on the use of nonverbal communication. That has definitely challenged me to adapt the way I practice. More than once I've had to tell a child, "I'm smiling underneath my mask. Although you may not be able to tell right now.” *(USA)*

Exhaustion became a recurrent theme as days blended into weeks and months, with little demarcation between days, a shift of roles, and virtual over in-person meetings: “I come home ‘dead’ every day from work…no rest for the weary” *(Israel)*. Even as many wrote of the privilege of caring for patients, the added responsibilities and shifting paradigms became increasingly arduous.

In the midst of momentous personal and professional dislocations for both providers and those under their care, there was solace to be found in whatever routines could be salvaged, including that of therapy:

One common refrain I hear from those who continue to see me is that they are not used to the new location and they're not used to the room that I'm now in. But they have found comfort and familiarity in the therapy tools that I have always used in a room that they have come to know and cherish. It is only when they see these and they see me that they realize that the room may have changed, the circumstances of our meeting are different, but the therapeutic relationship remains the same. In this time of uncertainty, the work we do together is a comforting constant. There is still what we have between us that remains unchanging. A bulwark against the unknown. For that one hour, we get stronger, together *(Singapore)*.

“Selfies with Zoom” became an unintended meme of the project: it was among the most common type of images submitted and reflected the pervasiveness of virtual communication platforms available to remain socially connected—and clinically active—while physically distanced. Virtual tools used in creative ways presented new opportunities to expand the reach of mental health supports, not only for patients and their families but also for colleagues:

In alliance with the mental health service of our practice, we created online support groups aimed at different care providers and open to any region of the country *(UAE)*.

For clinicians, the efficiencies inherent in seeing patients from the comfort of their respective homes was a welcome surprise:

we were able to follow families more closely, and that eliminated the difficulty in getting a suitable time to meet, to park, to balance so many other things that had affected the frequency of family meetings before the pandemic *(Israel)*.

The novelty of the experience started to wear off after so many hours in front of pixels rather than people. Zoom exhaustion led to a growing need to reconnect in person:

I miss people, I look for reassurance in other people. Although I deeply appreciate contemporary technology, I love to feel people around me. A person needs a person, simply to come closer. It’s not just our thoughts and our words: there is also that live presence of ourselves and everything we carry with us. All those congresses that we were traveling to, it was not so much to hear, so much as to feel presence in the moment; to belong to something so important to us *(Montenegro)*.

### 3.2 Outreach efforts

As crises often do, the pandemic brought out the best in some people. There were many inspiring and self-effacing images of psychiatrists and other mental health professionals dealing with the enormity of the crisis by going back to basics. Meeting people where they are came to take on a whole new meaning, as an admiring colleague noted on a fellow clinician’s commitment and fortitude:

He provides once monthly pro-bono consultation to a province in that small island. He brings his car and gets a two-hour ride in a roll-on/roll-off (RORO) ship. Then he drives four hours to reach his destination. He is the only psychiatrist who visits this province monthly and continued to do this regularly during this pandemic. There, he sees adults and children *(Philippines)*.

The pairing of mental health needs and basic survival hardships was hard to reconcile when facing individual patients and families:

I experienced a different story, that of poor people’s hunger. It seemed a cruel joke to tell those far less privileged fellow citizens about our well-intended mental health tips, rather than providing them with money or food to survive *(Bangladesh)*.

Local and regional outreach efforts were amplified through synergistic partnerships, such as collaboration with pediatricians or local authorities. Efforts were magnified electronically, and apart from physical and virtual outreach initiatives, there were upticks for treatment-seeking through already available resources:

On our online CBT program for child, teen, and parent anxiety, I was shocked to see that in that one week, our registrations were four-fold higher than usual. Clearly, I wasn’t the only one struggling. Some of the comments left online by parents shocked me and I felt a deep sadness for those who were struggling so much. Parents reached out to me personally, sending me videos of their clearly distressed children and how they were attempting to manage their stress *(Australia)*.

In a few paradigmatic instances, child and adolescent psychiatrists were strategically positioned to take the lead in country-wide outreach initiatives, as in the unique situation in which

a presidential technical committee on mental health was created. It was an honor being asked to direct it. This action is unprecedented, and watching the President speaking about the importance of mental health to the population has been a hopeful sign *(Chile)*.

### 3.3 Guild pride—and guilt

Mental health professionals wrote of their pride in taking on new roles in order to support colleagues working with COVID-positive patients. Notwithstanding a lack of necessary skills to adjust ventilator settings or provide ICU-level care, psychiatrists working in hospitals became valuable team members, even if not working at the “top of their license.” Being available to do “whatever is needed, psychiatric or not,” clinicians described

a heartening experience, for our team to have been given the priceless opportunity to support fellow medical colleagues as they helped one another and their patients, to ease their efforts as they made so many personal sacrifices *(Singapore)*.

In the frontline “trenches” of emergency room and inpatient hospital settings, service provision could not be switched over entirely to virtual care. The fraternity of these providers with “boots on the ground” shared a particular sense of mission and pride:

We showed up, we took the punches, we stayed open when people around us were closing up shop, we got sick, we got better, we looked after the vulnerable and marginalized kids we promised we’d always be here for, we didn’t just look after our own department and our own kids and families, we fanned out to wherever we were able to help in the hospital, we worked side by side with nursing, we cared for people who were dying, and we did it every single day and every single night *(USA)*.

Confronting so much illness and death on a daily basis, it was perhaps inevitable for health care providers to harbor feelings of inadequacy and guilt over not being able to do nearly enough to stem the tide. At an individual level, feelings of self-centered shame or guilt were not unusual:

I was ashamed of myself. Ashamed that I had only started to care about COVID-19 when it impacted my personal life…I am still working on making peace with the shame I felt at my initial insensitivity to COVID-19 and its impacts *(Pakistan)*.

I have been forcefully disconnected from my patients and their families. I know that I am an important figure in their lives and feel guilty of abandoning them…as if it was my fault…I wonder how they are doing… *(Spain)*.

At a group level, feelings of inadequacy partly stemmed from the framing of healthcare providers as “heroes,” an honorific that felt overused and misleading under the circumstances:

My thoughts were around the word “hero,” and all the talk around heroics so pervasive in the air these days. The term is usually applied to healthcare and “essential” workers. In some ways, I am aware that my team, and even I, have been considered “heroic.” Yet, the term feels inappropriate and uncomfortable. Countless families have not been able to afford the steep price of social distancing. It is these parents, mostly mothers, I'd imagine, staying at home, trying to make a life of normalcy and safety for their children, the endless days under the pandemic. It is they who are the heroes. The breadwinners among them, going out, taking risks to scrap a living. It is they who are the heroes *(USA)*.

Many clinicians were able to adapt their practices, almost overnight, to virtual platforms. Without the possibility of seeing patients in person—and without the need, at some level, to do so—a sense of dislocation crept up for some, as if being *of* the moment, but not fully *in* it.

We all sit comfortably in our homes at the end of the day. We have plenty of food, we have protective gear, we certainly have shelter. We have the internet. We complain of being for too long on Zoom calls. But what about the countless many who have limited or no connectivity? Zoom and the internet: such problems of affluence *(USA)*.

## Domain 4: Purpose (Table 4)

*And I submit that nothing will be done until people of goodwill put their bodies and their souls in motion. And it will be the kind of soul force brought into being as a result of this confrontation that I believe will make the difference.*

- Martin Luther King, Jr. [34]

**Table 4:** Purpose: themes and sample quotes

Theme	Sample quotes *(source country)*
4.1 Moving from pandemic to syndemic	The pandemic, if it is even imaginable, came to take a backrow seat to yet another crisis, a racial crisis long developing in this country. Four hundred years and more in the making. *(USA)*
4.2. From lamenting to embracing	People panicked. Stores were emptied out, shelves left bare –the reality once again of the social disparities were evident– those who could afford it stocked up for a war, but those who relied on social grants and a weekly wage could do nothing but look on helplessly as they were unable to buy anything, let alone more than usual to stockpile. *(South Africa)* Psychiatrists, like all physicians, need more than ever to speak out to the public to amplify good science while refuting nonsense. If we don’t, there are countless others, most of whom have never done science or actually cared for a patient, who are more than happy to fill the void. *(USA)*
4.3 Planning toward a better tomorrow	Following each forest fire, new green shoots emerge. COVID-19 has had the same effect, by providing us new opportunities that were not available to us before. *(Singapore)*

### 4.1 Moving from pandemic to syndemic

Blaming, racializing, and “othering” [35, 36] have long been maladaptive societal responses to unexpected turns of fate, including disease outbreaks. In the case of the COVID-19 pandemic, xenophobic tropes took hold by assigning blame for the pandemic’s origin. By nationalizing and anthropomorphizing the “Chinese” virus, populist demagogues sought to score political points rather than address a global emergency. As a result, Chinese and other Asian citizens living abroad often had to confront an additional, and unwarranted, level of animus:

The daily news reported Asian-Americans spat on and attacked with racial slurs. The “model minority” had led us to believe that working hard and not complaining would lead to peaceful coexistence. But even as we have become physicians, lawyers, engineers, and entrepreneurs, we continue to be the target of bias, violence, and racism *(USA)*.

*Syndemic*, a term first introduced by medical anthropologist Merrill Singer [37], is defined as a set of linked health problems involving two or more afflictions interacting synergistically. A syndemic contributes to an excess burden of disease or death in a given population, as in the case of COVID-19, in which health-related problems clustered by time, place, and race. The pandemic revealed underlying societal fractures, made evident by disproportionate death tolls not only across socioeconomic levels, but across racial—and racist—lines:

The pandemic would redefine what it meant to have access to care, as telehealth required having access to stable internet, highlighting the staggering inequalities in health care access, with higher COVID-related mortality among minority populations. The pandemic forced a long overdue reckoning with racism *(USA)*.

The inexcusable overrepresentation of minorities, the indigent and the poor among the dead. And yes, an unacceptably high number of Blacks among the dead. This image reminds me of those in the shadows, those overlooked, those brown and Black faces that we have not served right *(USA)*.

I've fluctuated between gratitude for this time with my children and our safety, being able to have a job and work remotely, and a deep sadness and insecurity for all Americans of color…I have reticent gratitude that my kids live in safety, but deep rage and sadness that not all kids of color can *(USA)*.

### 4.2 From lamenting to embracing

We found it helpful to divide the many images and reflections we received that related to social inequities into two conceptually related yet different categories. The first, which we term *lamenting*, refers to the overwhelmed witnessing that characterized the earlier months of the pandemic, as the calamity started to become apparent, but before any organized mobilization was in place. Lamenting is not to be construed as a passive phase, as it is a *receptive* one: in order to lament one has to be able of opening up, in distinct contrast to disillusionment, emotional unavailability, or lack of sensitivity. It is an open phase and a precondition for the next stage of mobilization. A pervasive sense of paralysis and disorientation characterized the lamenting stage:

This public health crisis is exacerbating existing vulnerabilities, access to care services, and gross inequalities, particularly for young women and girls *(Chile)*.

With their daily hand-to-mouth sustenance, I wonder who is feeding their children and how many of them have gone to bed hungry for days. I think about the systemic racial inequities around me, the state-sanctioned murder of Black people, the Latinx children caged at the borders, the internment camps in China. And, all the while, the red death claims more victims. A storm ravaging a storm. And my heart is broken *(USA)*.

It seems that the “voice” of children is yet again drowning in the fear and priorities of adult society. Children are so easily forgotten *(Poland)*.

As the weeks dragged on, clinicians actively engaged in the different adaptations outlined in the *embracing* theme, which came to redefine what they could do as mental health professionals. As they confronted the social inequities laid bare by the pandemic, many took to activism and mobilization with renewed vigor. Some efforts were modest, local and meaningful, as when “some colleagues and I organized volunteer programs to a kindergarten.” *(Vietnam)* In other instances, it was youth themselves who set the examples for innovative action, as witnessed by a child psychiatrist who was

stunned into silence when I learned that my 16-year-old son was part of a veritable underground army of formerly nerdy computer types, now become heroes of sorts by producing in countless basements throughout the nation what the country itself was not able to provide: PPE…The following day, he took a large box to the Veterans Hospital and donated equipment to its frontline staff. He was keen on having the equipment go to the “unsung heroes”: the cleaners, janitors, phone operators, nursing assistants, and countless others keeping the healthcare system ticking *(USA)*.

Some examples were nothing short of heroic, as when a group of psychiatrists addressed the most basic of mental health priorities—securing food and shelter—for a population doubly hit by the pandemic and by the unprecedented flooding of low-lying coastal regions:

He went with some of his friends to the remotest islands to distribute relief. Along with basic food items they distributed essential medicines, clothes and handwash. A surprise request from the locals was for mosquito nets to be used for protection against snakes while sleeping at night *(India)*.

Even as the pandemic raged on with no end in sight, such acts of goodwill and selfless collaboration gave a much-needed sense of hope for enduring effects in the long recovery ahead:

It is not clear where we will end up, it is not even clear where we are today, but the stirrings of social justice that have been so painfully and magnificently evident across the land do give me hope *(USA)*.

The black plague heralded the Renaissance; surely we are looking at another period of enlightenment ahead *(Pakistan)*.

### 4.3 Planning toward a better tomorrow

Visual and textual contributions during the first 4 months of the pandemic were snapshots, in real time, of a crisis actively unfolding. There was no clear sense of finality or of a timeline for a return to normalcy. In that temporal context, anticipation for the future was as full of hope as it was uncertain. One participant wrote of

the medical students that I am privileged to teach, still fierce and passionate, determined to leave the world better than they found it, and my friends and colleagues, still with the embers of hope that goodness and justice will prevail *(USA)*.

There was a growing sense that clinical practice would never return entirely to its pre-pandemic shape. For example, as a field we would be hard pressed to give up on some of the inroads so rapidly made in telepsychiatry or in the virtual access to remote locations or reluctant patients. Paradoxically, and thanks to electronic means for communication not available during last century’s pandemic, the enforced physical distancing may have only heightened the precious value of social engagement at both personal and professional levels:

the concept that *life is with others* –as our dear Professor Donald Cohen said in his eponymous book [38]– has become more meaningful and precious for me these days. I predict that enhanced connection, interdependence, and collaboration across near and far will be the positive corollary and lasting impact that the pandemic will have on children and on child and adolescent psychiatry *(Spain)*.

## Discussion

Photo-elicitation offered a unique vantage point on the experiences of child and adolescent mental health providers working around the globe during the early months of the pandemic. We turn to address three aspects of the project: (1) our research methods of choice; (2) some of the key themes we found, with particular emphasis on the inequalities and social justice challenges laid bare by the pandemic; and (3) opportunities for positive change to emerge from a crisis still actively unfolding. We close by considering our study’s limitations and future opportunities.

### Encapsulating the pandemic

In an effort to get vivid reflections of child and adolescent mental health providers’ pandemic experiences, we were able to tap into the ubiquity of smartphones with digital cameras, voice memo recorders, email, text, and other communication apps. The ease to obtain and distribute these “field notes” permitted us to document the early and disorienting first 4 months of an unfolding global crisis. The majority of submitted images, reflections, and voice memos were candid, unpolished, and draft-like in form. Photo-elicitation has been described as able to provide “a path to the ‘fusion of horizons,’ or a broadening of one’s own horizon of knowledge by opening up the [reflection] to the content and communicative potential of images and the linguistically negotiated interpretations, descriptions and meanings they invoke.” [12]. Indeed, we posit that images succeeded in their ability to tap into deeper elements of consciousness than words alone would have [39]: our use of photo-elicitation went beyond simply gathering *more* information to evoking a *different kind* of information [40].

Uncurated and raw, the combination of these visual and written materials offered a window into highly personal instances of what Donald Schön’s classic work has termed *reflection in action* [41]: the metacognitive understanding of what is being done while doing it, and that allows behavioral modulation and course correction in real time. Apart from documenting and providing first-person testimony for an unheralded time, a collection of this kind may prove useful later on, once better contextualized in time:

We write these “reflections” in the moment, as the impacts of the pandemic unfold around us daily. We are all living it right now. When it is over, we will look back and reflect upon it and with the benefit of hindsight, might make normative judgements regarding what we ought to have done and what might have been best at a certain time [42].

Nicol and colleagues have made an entreaty to the field to chronicle the extraordinary threat and its effects on mental health, and specifically, to embed qualitative approaches into ongoing research efforts [43]. Through a qualitative approach, we have sought to tap into the granularity of mental health clinicians’ everyday experiences in both their personal and professional lives. The boundaries between the two gradually blurred, just as they did between weekdays and weekends, or between home and office. Our interspersal of three epigraphs throughout this academic manuscript can be construed as yet another echo of such blurring under disorienting conditions, an approach that has been movingly and effectively applied by Ryan Petteway “as creative praxis to analyze and reflect on our present moment in relation to public health pasts and to raise questions about public health research, education, and data futures.” [44].

### Global snapshots of an overstrained profession

The pandemic made painfully evident, on two different planes, stark differences in resources, approaches, clinical care, and morbidity and mortality. As we launched the project, we were strategic and deliberate in approaching one of those two planes—that of global discrepancies.

By the time we started data collection in April 2020, several thousand articles on the COVID-19 pandemic had already been published since the Sars-CoV-2 virus’s first emergence in late 2019. This large body of scholarship primarily represented the work and perspectives of researchers in China, India, the United States, Australia, and several European nations. There was a scarcity of articles on the pandemic produced by researchers in low- and middle-income countries (LMICs). Many LMICs, particularly on the African continent, had not yet published a single article on the pandemic [45]. This imbalance in research productivity suggested that the existing literature was representative of the needs and views of only a portion of the world. We thus sought to be as widely inclusive and representative of the global experience. And even as the final geographic distribution of our sample was disproportionately representative of the United States and Europe, it did span 54 countries, including 23 LMICs. It was important to us, in our aim to better represent and understand the experiences of child mental health professionals globally, that our sample be truly representative in its spread. We were reasonably successful in this effort, allowing us to conduct thematic analysis with a meaningfully diverse data set.

We were admittedly less intentional in prospectively addressing the second plane—that of the jarring intersection between COVID-19 and social injustice in general, and structural racism in particular, even as it ended being one of the most common, discomfiting, and heartfelt themes. As a case in point, the syndemic section of our results is the only one in which all of our quotes come from the United States. This is likely a reflection of the specific time window of our study: A series of highly publicized events in the US, epitomized by the on-camera murder of George Floyd by Minneapolis police officers on May 25th, *pictured* long unseen atrocities and served as a flash point for the protests and mass demonstrations that followed [46].

Just as the pandemic is a global phenomenon, so too is racism. Racism and social injustice have emerged as another pandemic within the pandemic. In the early weeks of the pandemic, government officials touted COVID-19 as the “great equalizer,” as no one is immune to the virus: “Viruses do not discriminate, but society does.” [47] COVID-19 is indeed indiscriminate in its transmission, but its propagation within society became steeped in structural racism and health disparities that led to the abhorrent and disproportionate impact on marginalized racial groups in the US [48, 49] and globally [50, 51].

Physical distancing emerged as a scientifically proven strategy to “flatten the curve” and slow spread of the virus. And yet, it was a privilege to which low-wage and “essential” workers—disproportionately people of color—were unable to adhere, either because of being mandated to work or because of fear of lost wages or permanent unemployment [49]. The impact on the children and families of those marginalized workers will continue to reverberate during the hoped-for long return to normal [4]. Applying frameworks such as intersectionality and critical race theory will be necessary in order to examine the ways existing power structures and underlying racial inequities will produce both short- and long-term consequences [49]. Many, but certainly not all of the racial tensions we learned about came from the Unites States. We are cognizant of the risk of overgeneralization, and following decolonial scholarship, critique the export of high-income frameworks to non-Western contexts. Racism is not unique to the US , and to borrow Chakrabarty’s term, we want to be mindful not to “provincialize” the American experience [52].

### Flickers of opportunity amidst an unfolding crisis

COVID-19 has presented an opportunity for psychiatry to extend and reimagine its reach, for clinician-scientists to move “from the role of neutral observer to one of engaged contributor,” [43] understanding that this shift is not only the right thing to do during a public health crisis, but one that will strengthen the field’s relationship to the public, which has historically lagged behind other medical professions [53]. We join Moreno and colleagues in the sentiment that

The interconnectedness of the world made society vulnerable to this infection, but it also provides the infrastructure to address previous system failings by disseminating good practices that can result in sustained, efficient, and equitable delivery of mental healthcare [54].

It remains to be seen, and will be contingent on us, whether as a field we will rise to the occasion and capitalize on the opportunity to improve mental health services that the pandemic crisis has abruptly opened. Mental health professionals from around the globe shared some of the unique contributions they have made during the pandemic, from utilizing social media as an educational tool to promote emotional wellbeing, to supporting colleagues caring for COVID-positive patients, to providing direct care under new virtual paradigms, to reorganizing entire systems of care. It is vital that the resourcefulness, collaboration, and ingenuity demonstrated during the pandemic be carried forward, scaled, and accompanied by funding so we that can respond to what has already been deemed a “looming mental health crisis.” [55, 56].

We are starting to see some of the negative consequences of COVID-19 on the mental health of children, adolescents, and their families, and as the pandemic persists, sequelae will likely only increase in severity and magnitude. There is considerable and historic underinvestment in mental health across the globe, especially in LMICs [57], despite the burden of mental illness globally [58]. Open before us is an opportunity to build a better system, one that is embedded in sustainable adaptations of mental health delivery and “informed by ethics- and rights-driven considerations” [54]. Stakeholders, decision-makers, clinicians, and consumers must come together in a shared commitment to ensure that adequate mental health services are provided in order to address current and future needs.

## Limitations and future opportunities

We recognize several limitations to our study: (1) Even if adequate for a qualitative study of this type, our sample size was modest. Still, we consider having done well on the trustworthiness of our report as reflected by the qualitative constructs [59, 60] of *credibility*, or the plausibility of our descriptions being recognized by its participants, and of *transferability*, or the applicability of findings to other settings and programs; (2) We focused exclusively on care providers; by leaving out the direct mental health experiences of children and their families, particularly those of families of color or those with limited access to broadband internet, we missed an opportunity to "center on the margins" by incorporating the perspectives of socially marginalized groups, rather than just those of people belonging to a dominant race or culture [61]; (3) Despite a reasonable response from LMICs, there are broad swaths of the globe, particularly in Africa, that are not well represented in our data. This skewed representation is partly related to our snowball sampling, given that mental health professionals in LMICs may have more limited access to a larger online social network, or face community bias against or government restrictions around this candid research approach (as reflected by a handful of participants who retracted their initial agreement to participate); (4) We constrained, by design, the dates of data collection. As such, our findings are relevant to a very specific time interval; our approach could be fruitfully applied to later stages of the pandemic; and (5) Even as we did our due diligence to attend to the specificities of each cultural context, we recognize, as researchers working and thinking primarily within the US and Europe, that our analysis of the pandemic is shaped by our own contexts.

## Conclusion

Photo-elicitation provided a disarming and efficient means to learn about individual, regional, and global similarities and differences regarding the professionals charged with addressing the mental health needs of children and adolescents during 4 early months of the COVID-19 global pandemic. These findings may help inform practice changes in post-pandemic times: by thoughtfully incorporating novel technologies for clinical care and distant learning; by providing a new impetus for networking opportunities ranging from the regional to the global; by helping reimagine clinical outreach possibilities—using web-based means for those with access to the internet, and innovative community-based partnerships for those without; and by reinvigorating a sense of purpose, a commitment to equitable care, and an adherence to ethical, socially progressive, and anti-racist principles.

## Supplementary Information

**Additional file 1: Appendix S1.** List of countries with at least one photo-elicitation contribution to the study.

**Additional file 2: Appendix S2.** Sample of images examplifying domains and themes.

## Data Availability

The datasets obtained and analyzed during the current study are available from the corresponding author on reasonable request.
