# Correction to: Viral time capsule: a global photo‑elicitation study of child and adolescent mental health professionals during COVID‑19

**DOI:** 10.1186/s13034-021-00366-6

**Published:** 2021-02-27

**Authors:** Olivia D. Herrington, Ashley Clayton, Laelia Benoit, Cecil Prins-Aardema, Madeline DiGiovanni, Indigo Weller, Andrés Martin

**Affiliations:** 1 Yale School of Medicine, New Haven, CT USA; 2 Department of Psychiatry, Yale School of Medicine, New Haven, CT USA; 3 Public Health and Sociology, Inserm (Institut National de La Santé Et de La Recherche Médicale) and CESP (Centre de Recherche en Epidémiologie et Santé Des Populations), Paris, France; 4 GGZ Drenthe (Geestelijke Gezondheids Zorg: Mental Health Care), Beilen, The Netherlands; 5 Center for Educational Development and Research in Health Sciences (CEDAR), University Medical Center Groningen, Groningen, The Netherlands; 6 Bioethics Program, Harvard University, Cambridge, MA USA; 7 Child Study Center, Yale School of Medicine, 230 South Frontage Road, New Haven, CT 06520-7900 USA

## Correction to: Child Adolesc Psychiatry Ment Health (2021) 15:5 https://doi.org/10.1186/s13034-021-00359-5

Following publication of the original article [1], the authors identified errors in affiliation and in the conclusion section. The following corrections are presented with this erratum:

In the conclusion, the first sentence should read, “Photo-elicitation provided a disarming and efficient means to learn about individual, regional, and global similarities and differences regarding the professionals charged with addressing the mental health needs of children and adolescents during 4 early months of the COVID-19 global pandemic”.

The original article [1] has been updated.
