# Supplementary material for: Viral time capsule: a global photo-elicitation study of child and adolescent mental health professionals during COVID-19
Source: Child Adolesc Psychiatry Ment Health. 2021 Feb 2;15:5. doi: 10.1186/s13034-021-00359-5 (PMC7852478; doi:10.1186/s13034-021-00359-5)
Supplement: Supplementary file 1 — Additional file 1: Appendix S1. List of countries with at least one photo-elicitation contribution to the study. [file 13034_2021_359_MOESM1_ESM.pdf]

Appendix 1. List of countries with at least one photo-elicitation contribution to the study.

**Continent** (number of countries)	**Country**	**World Bank classification**
**Africa** (6)	*Cameroon	LMI
	Egypt	LMI
	Nigeria	LMI
	Rwanda	LI
	*South Africa	UMI
	*Tunisia	LMI
**The Americas** (10)	*Barbados	HI
	*Brazil	UMI
	*Canada	HI
	*Chile	HI
	Colombia	UMI
	*Dominican Republic	UMI
	Ecuador	UMI
	Mexico	UMI
	*United States	HI
	Uruguay	HI
**Asia** (17)	*Bangladesh	LMI
	China	LMI
	Hong Kong	HI
	*India	LMI
	Indonesia	LMI
	*Israel	HI
	*Japan	HI
	* Pakistan	LMI
	Philippines	LMI
	Qatar	HI
	Saudi Arabia	HI
	Singapore	HI
	South Korea	HI
	Taiwan	HI
	Turkey	UMI
	UAE	HI
	Vietnam	LMI
**Europe** (19)	Albania	UMI
	Andorra	HI
	Austria	HI
	Cyprus	HI
	France	HI
	Germany	HI

	Greece	HI
	*Ireland	HI
	Lithuania	HI
	Montenegro	UMI
	Netherlands	HI
	*Poland	HI
	Russia	UMI
	Slovakia	HI
	*Spain	HI
	Sweden	HI
	Switzerland	HI
	Ukraine	LMI
	United Kingdom	HI
**Oceania** (2)	Australia	HI
	New Zealand	HI

* Image from countries denoted with an asterisk are included in Appendix 2.

Note: Based on contributions from 188 entrants, spanning 54 countries.

Note: World Bank classification is based on 2020 data; LI = low income; LMI = low middle income; UMI = upper middle income; HI = high income.
