# Supplementary material for: Viral time capsule: a global photo-elicitation study of child and adolescent mental health professionals during COVID-19
Source: Child Adolesc Psychiatry Ment Health. 2021 Feb 2;15:5. doi: 10.1186/s13034-021-00359-5 (PMC7852478; doi:10.1186/s13034-021-00359-5)
Supplement: Supplementary file 2 — Additional file 2: Appendix S2. Sample of images examplifying domains and themes. [file 13034_2021_359_MOESM2_ESM.pdf]

1.Place

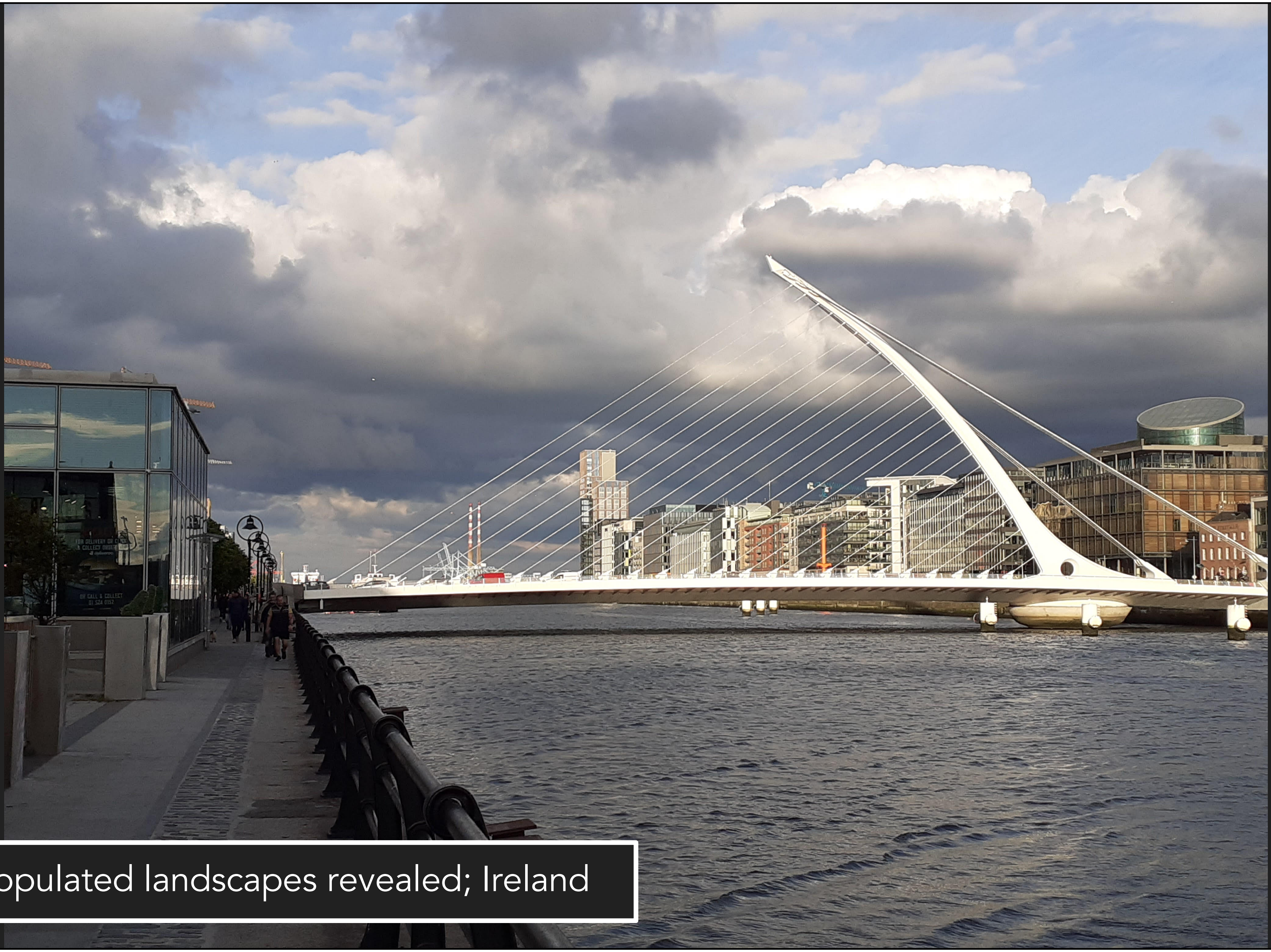

1.1 Unpopulated landscapes revealed; Ireland

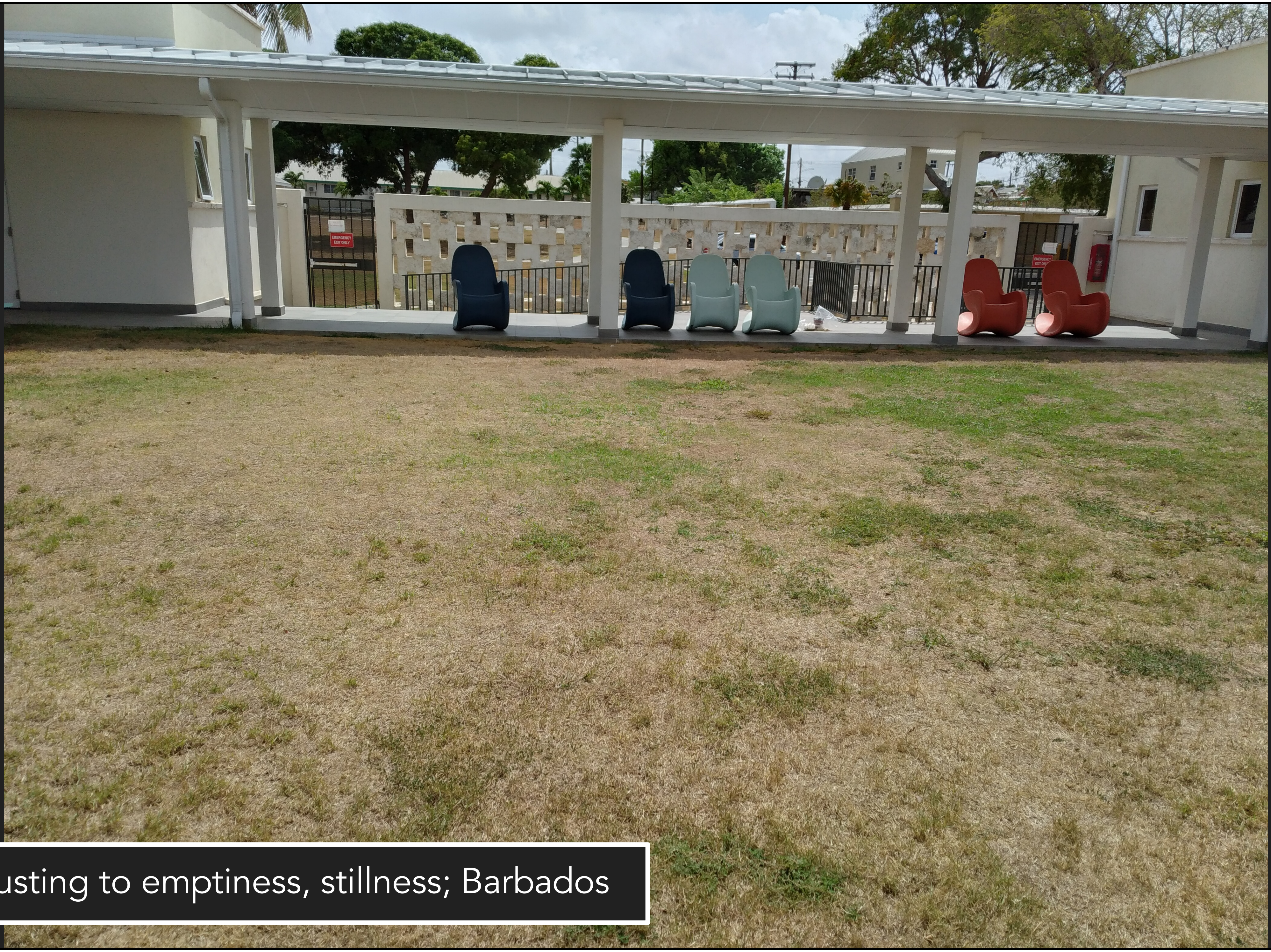

1.1 Adjusting to emptiness, stillness; Barbados

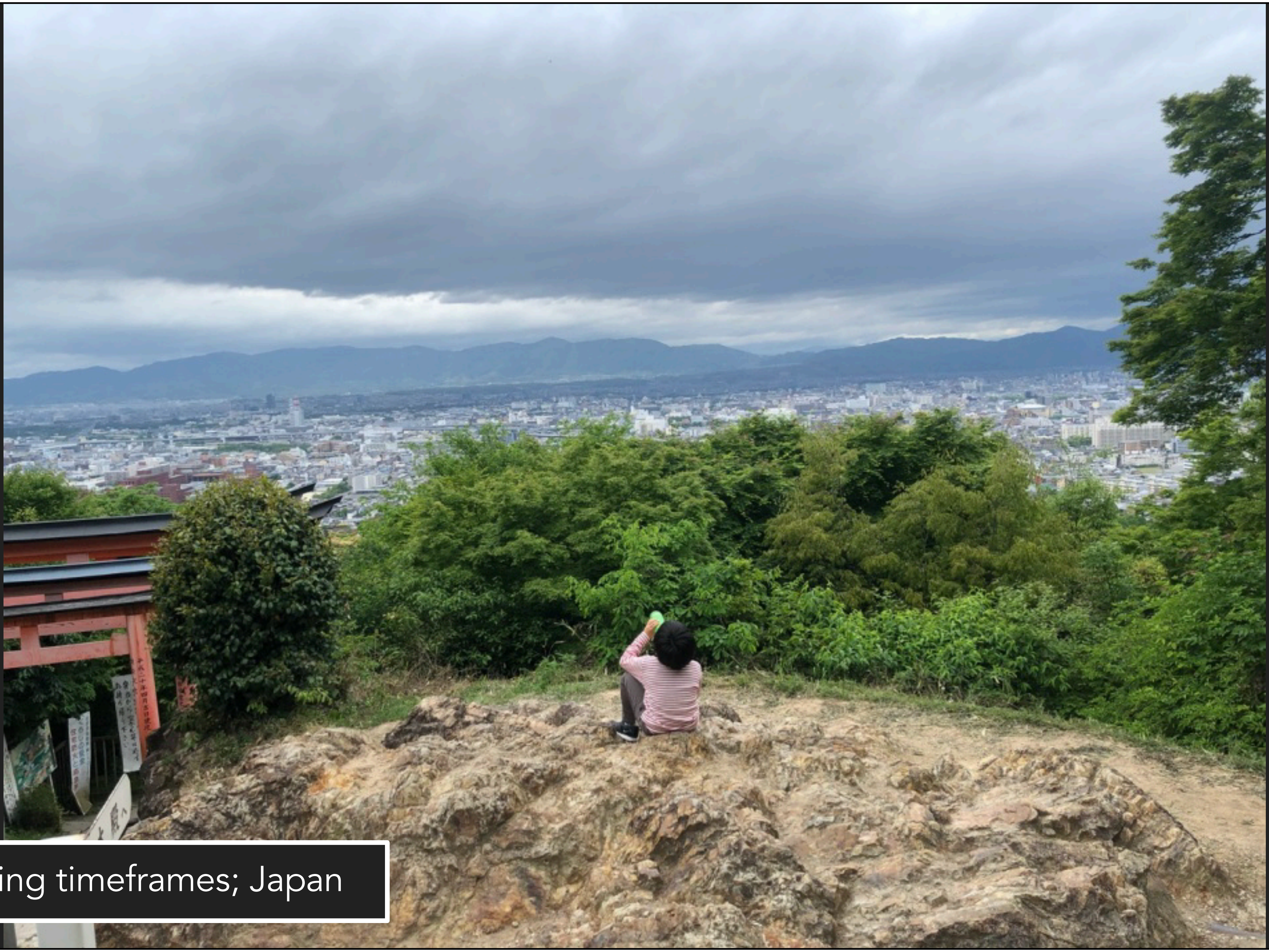

1.2 Shifting timeframes; Japan

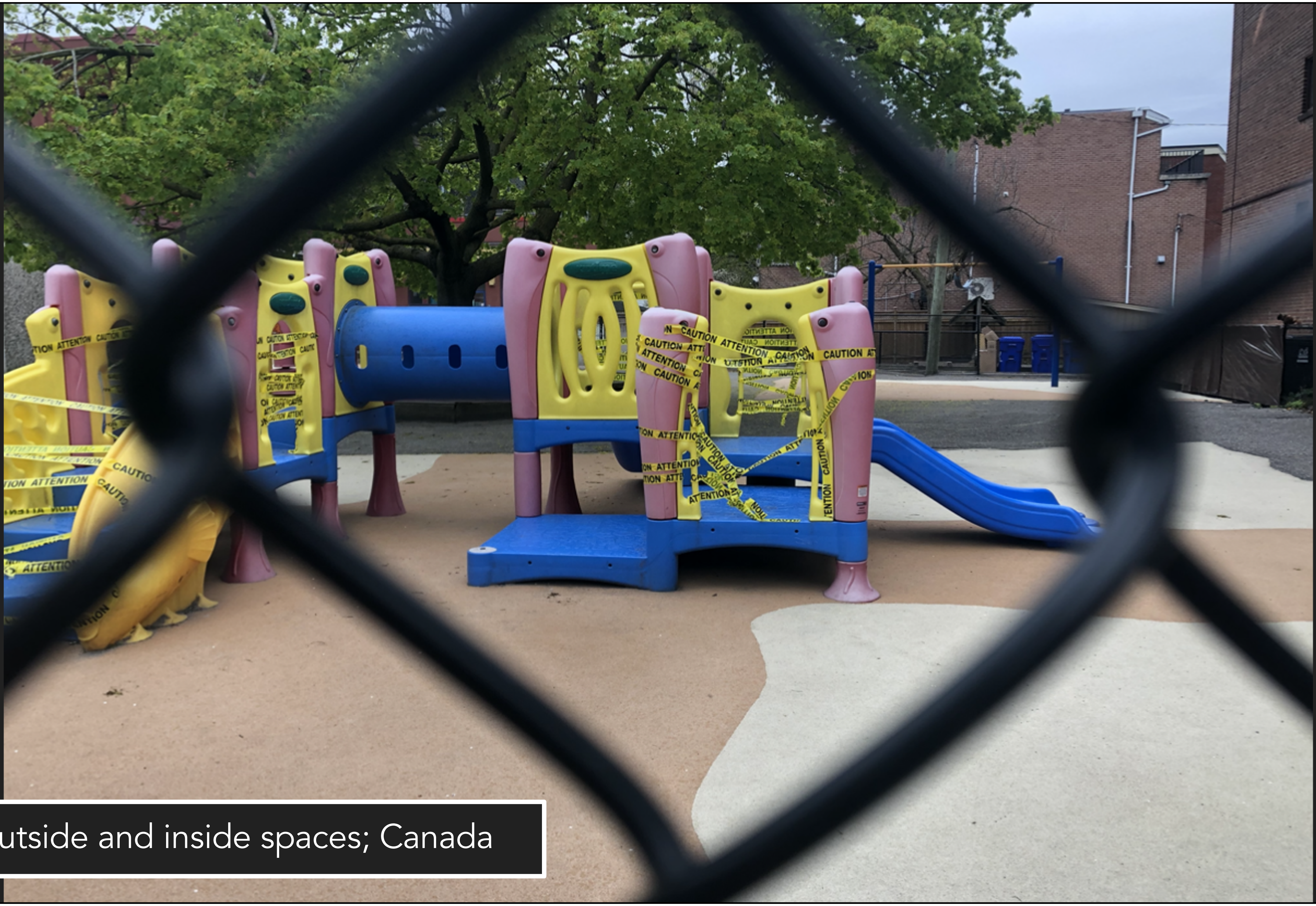

1.3 Outside and inside spaces; Canada

1.3 Blending of personal and professional spaces; Bangladesh

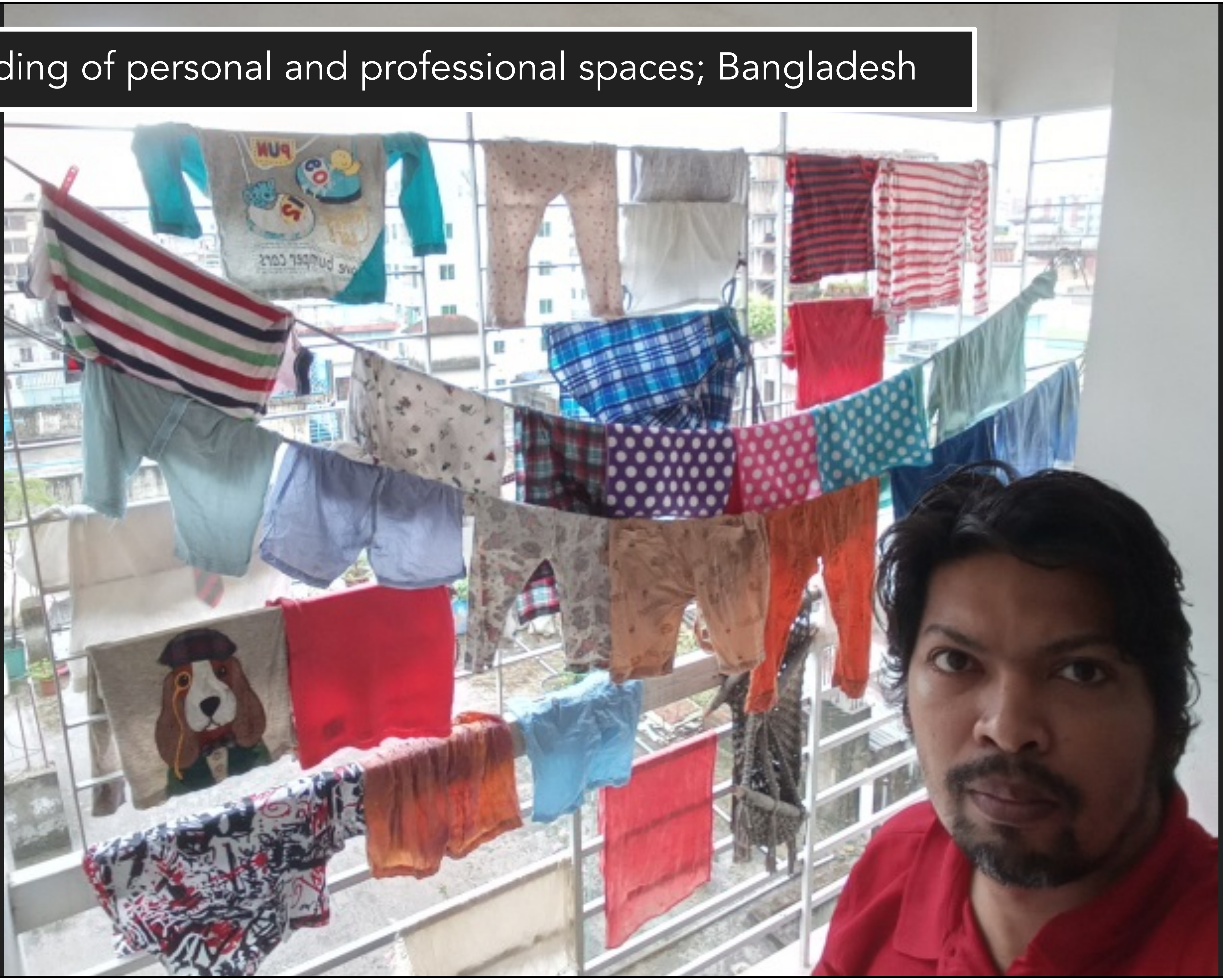

2. Person

2.1 Disruption to life rhythms; Spain

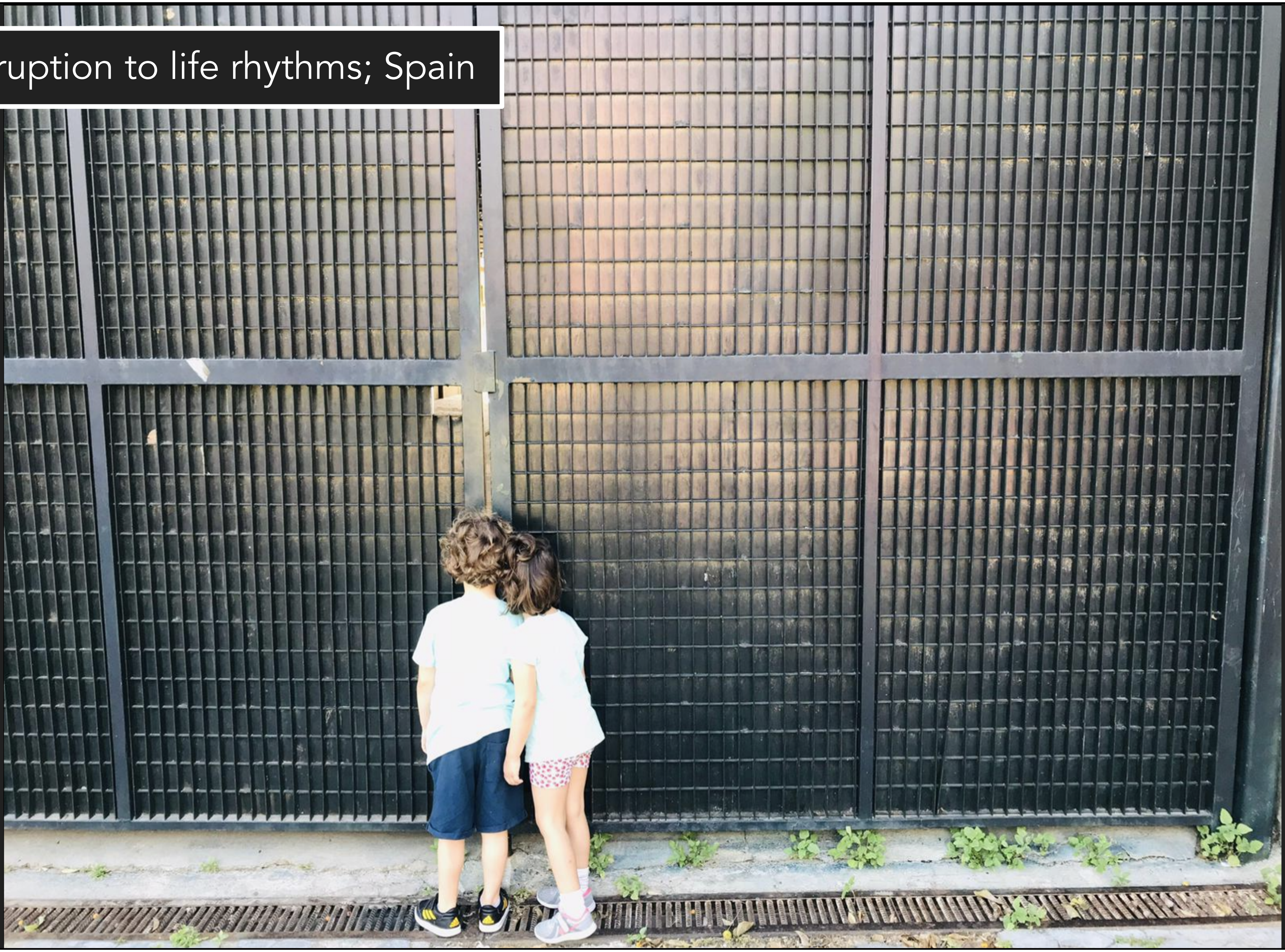

2.1 Family impact; Brazil

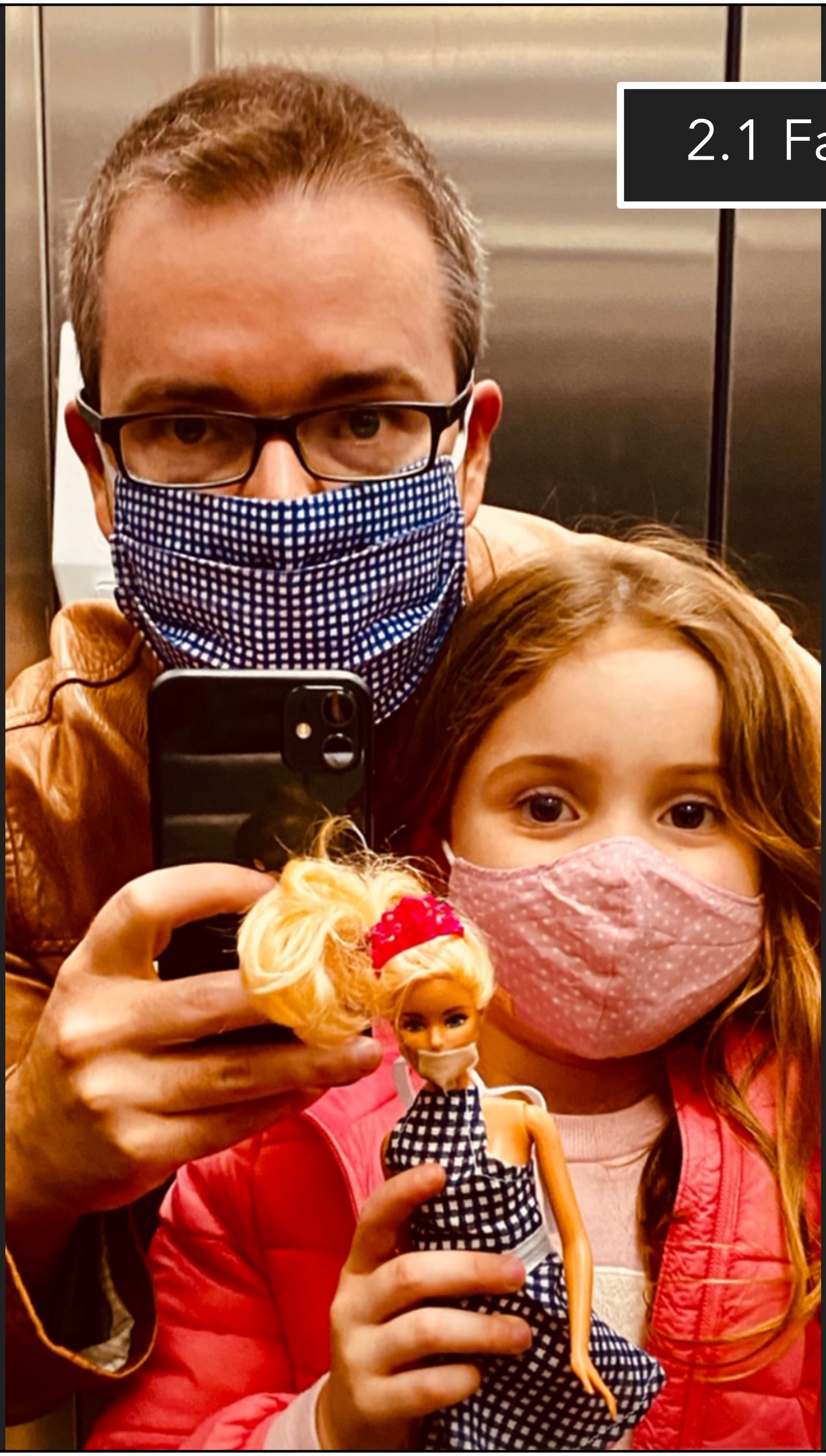

2.2 Emotional toll; Pakistan

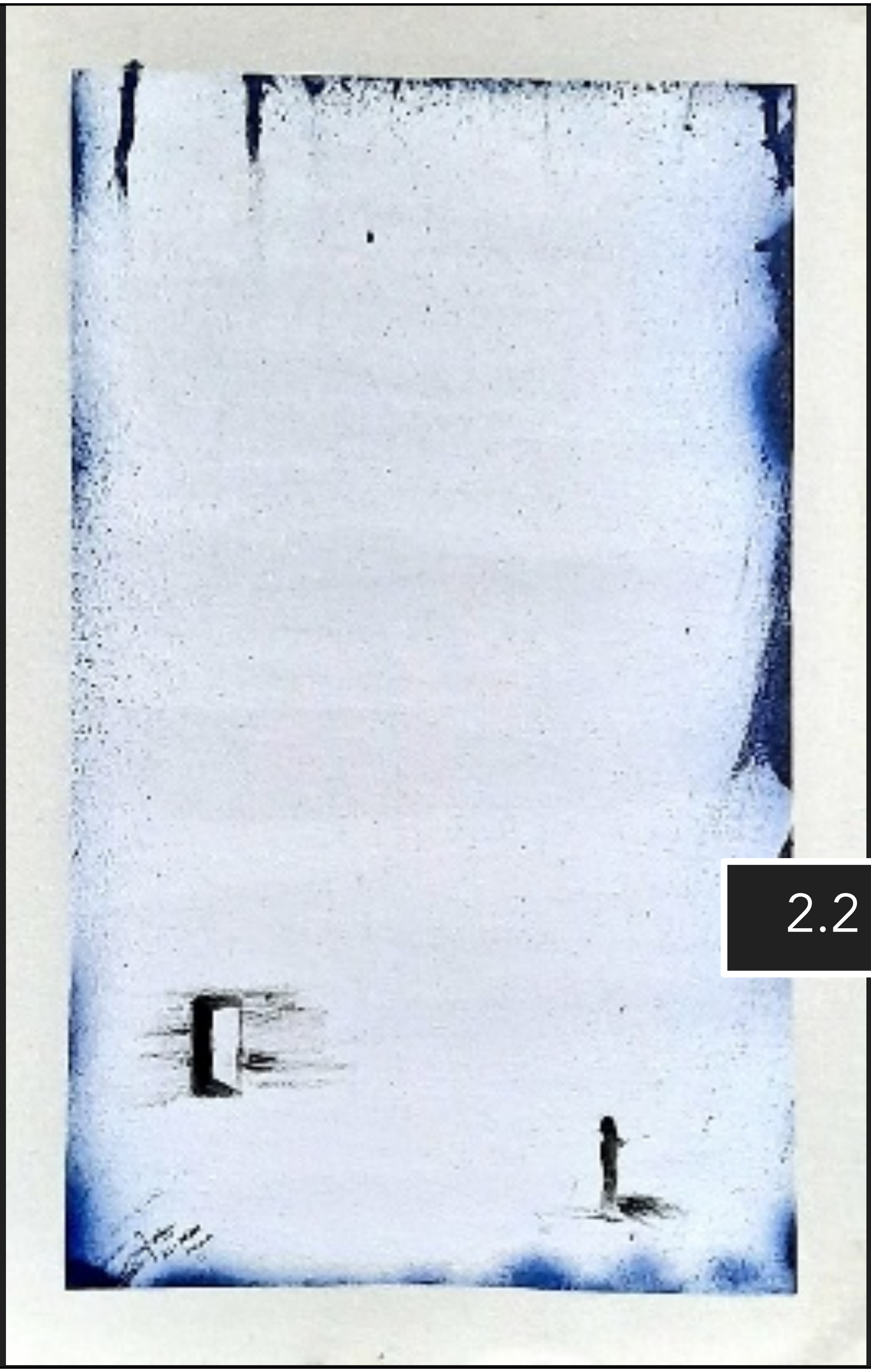

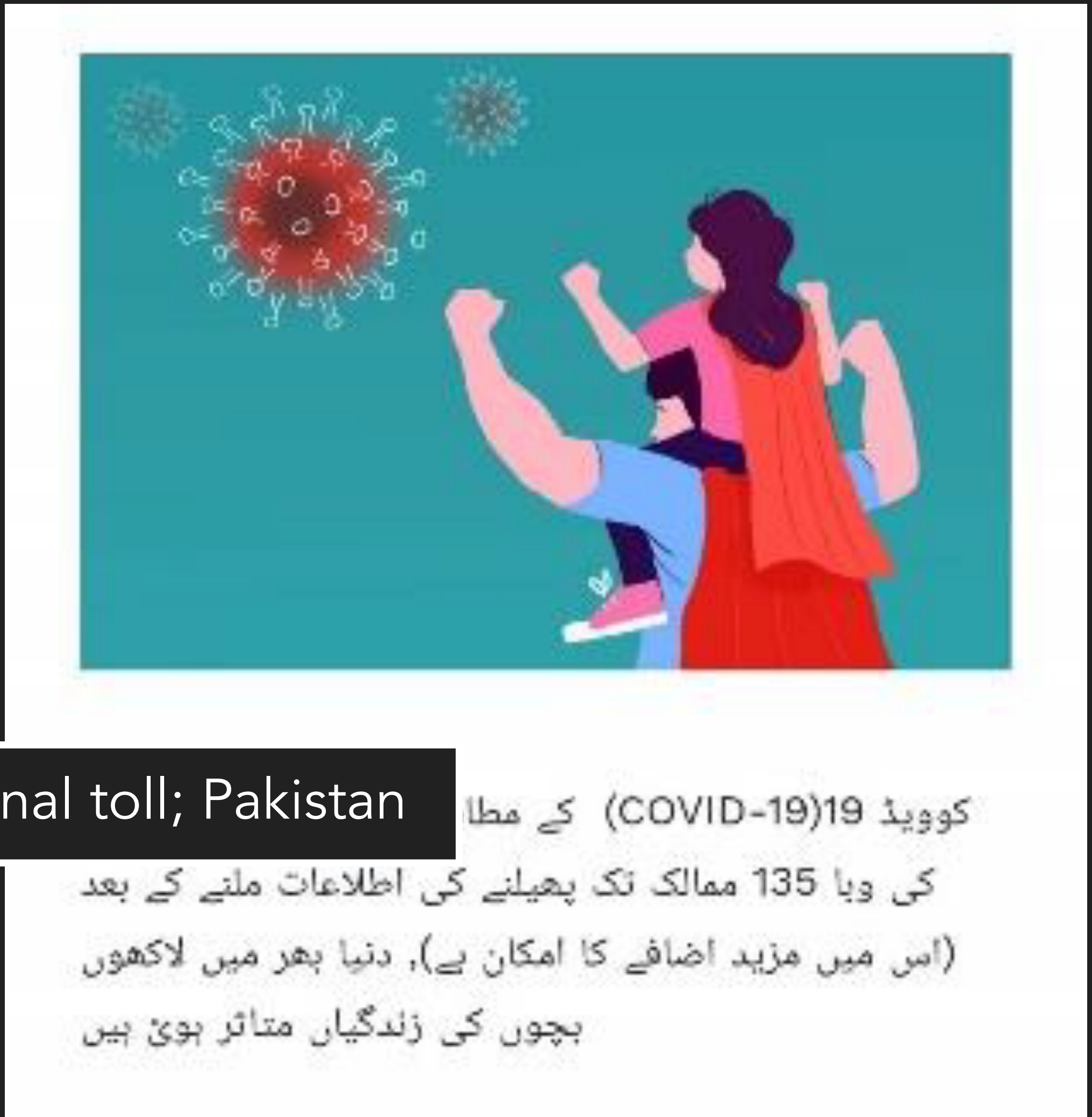

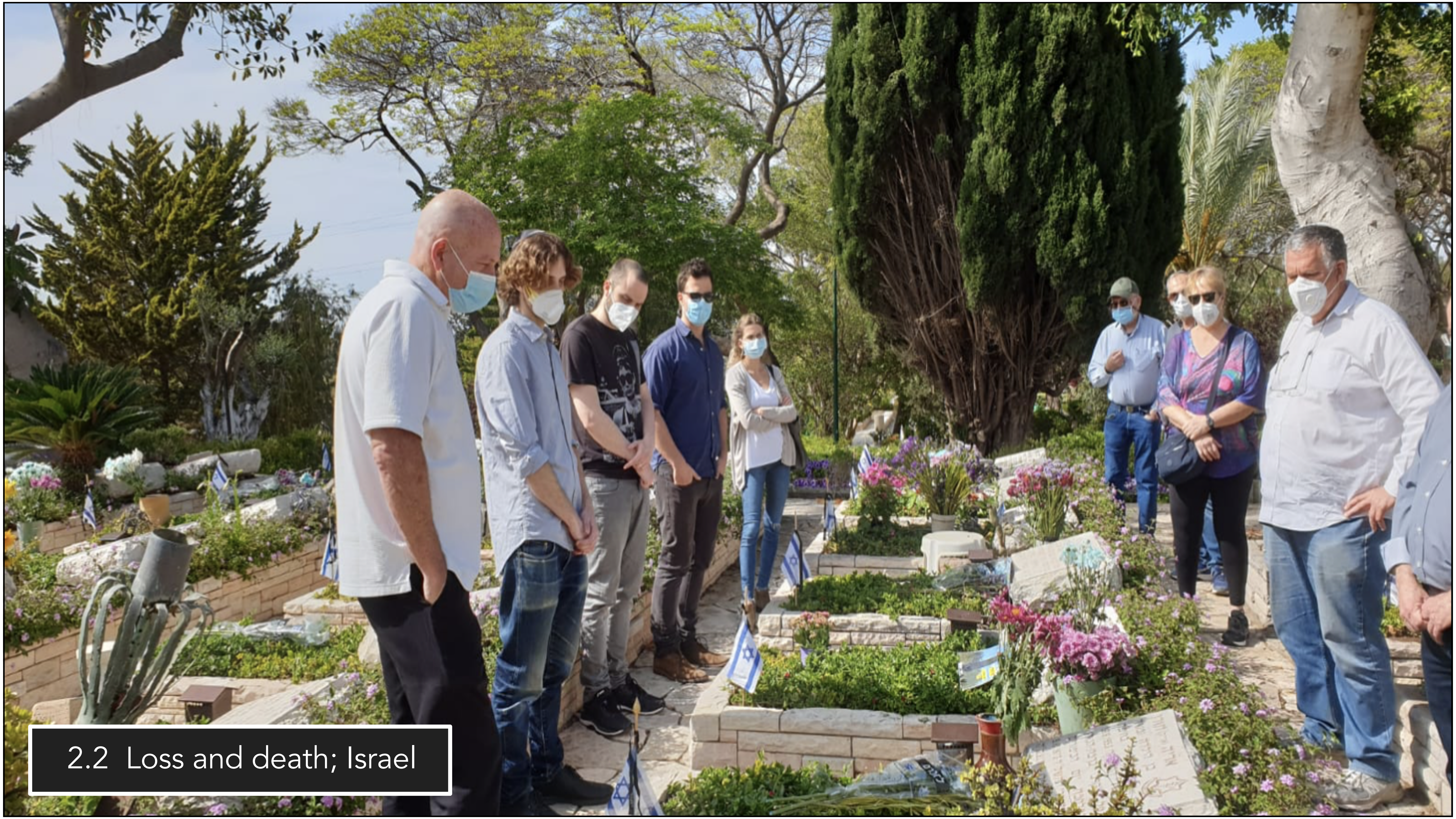

2.2 Loss and death; Israel

2.3 Positives of the pandemic; USA

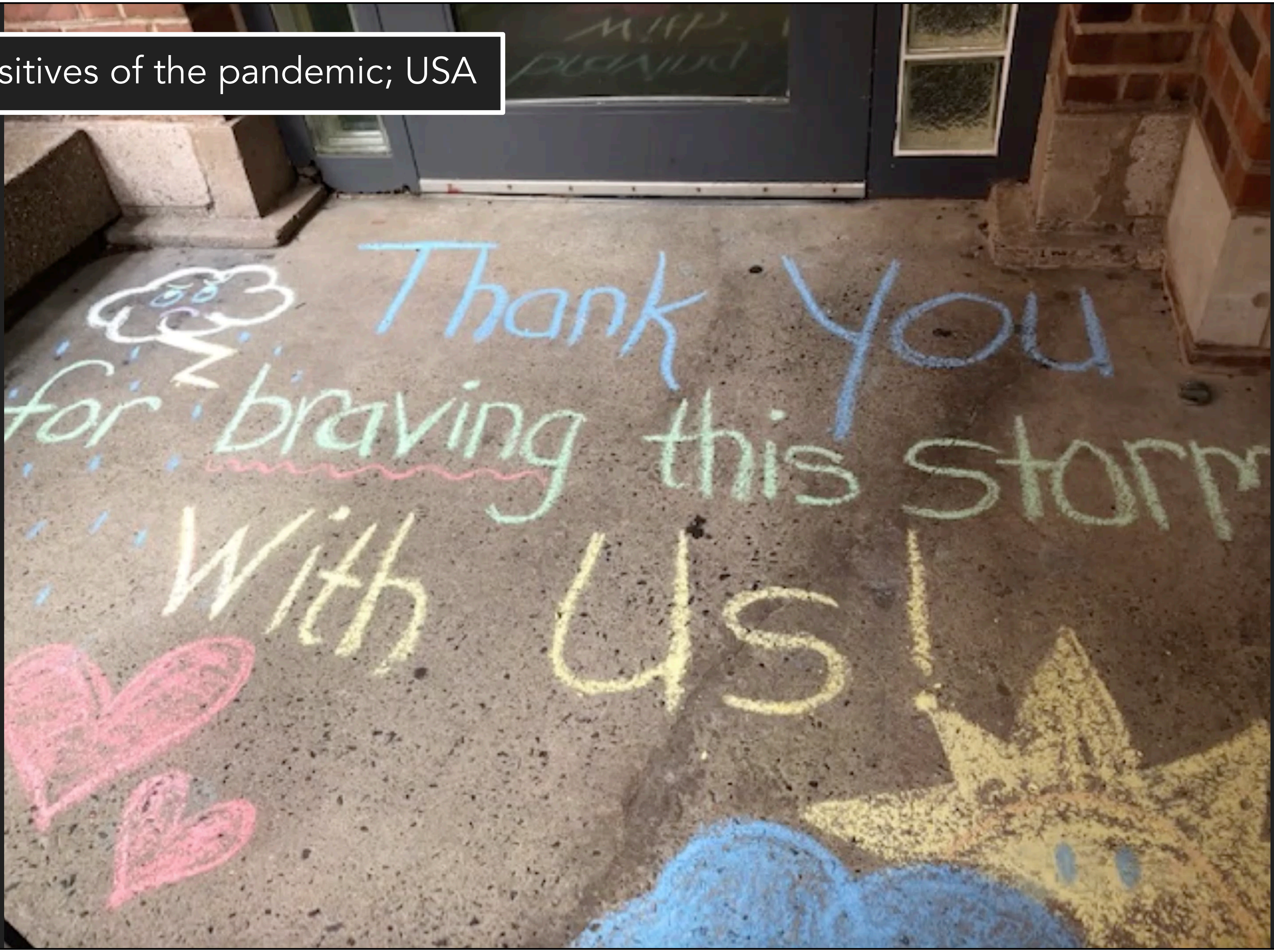

3. Profession

3.1 Virtual opportunities and limitations; Tunisia

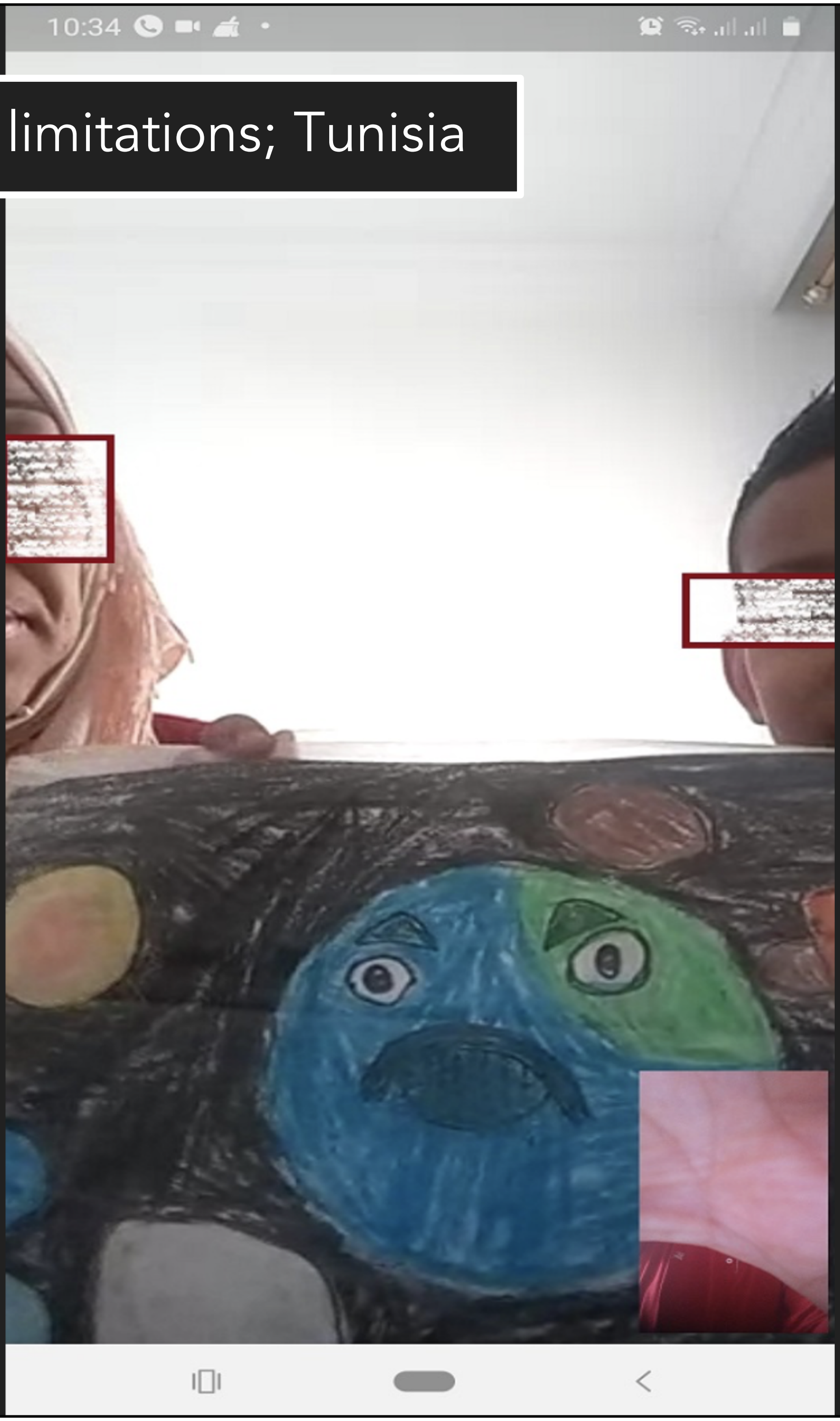

3.1 Changing practices; Dominican Republic

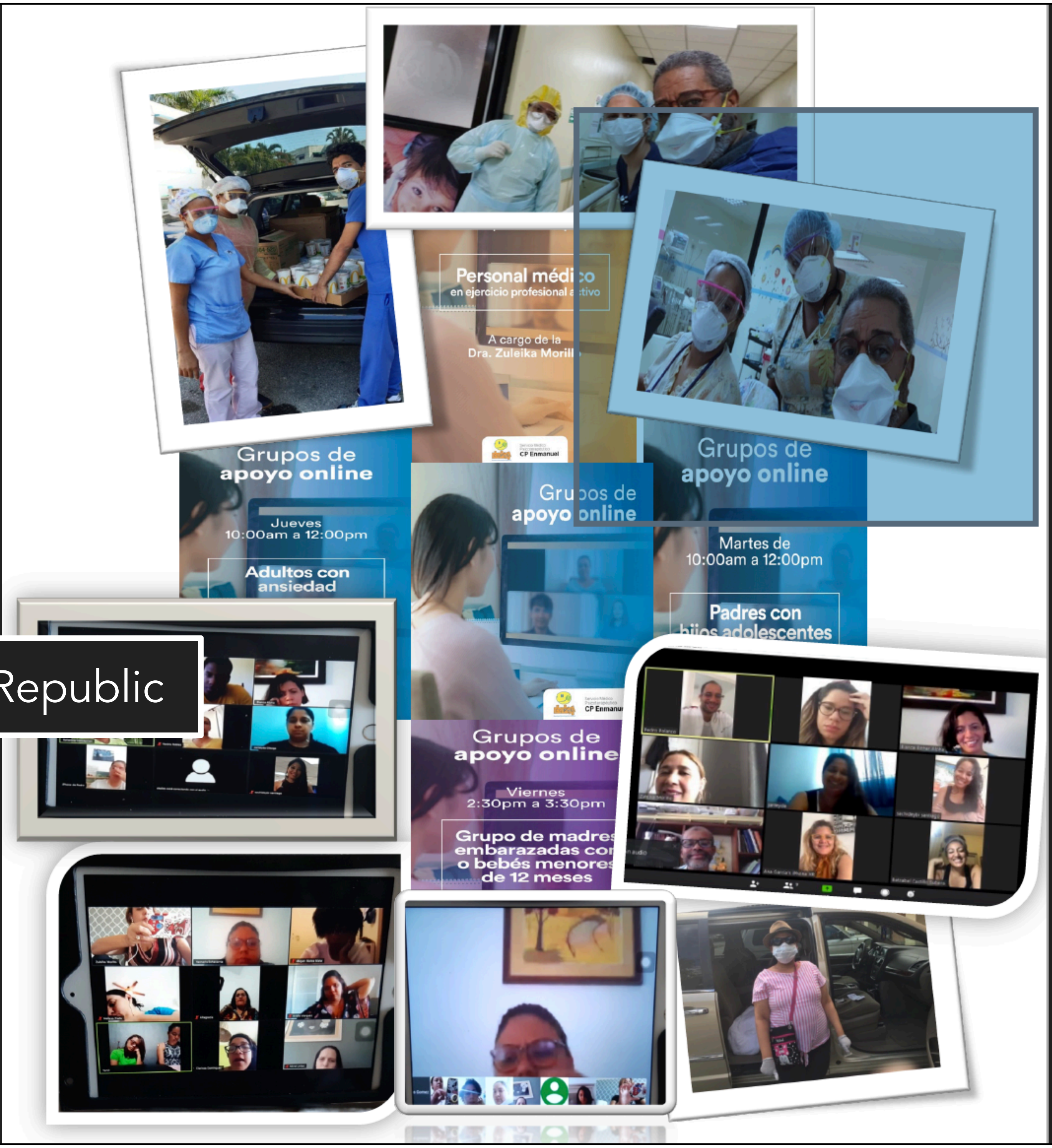

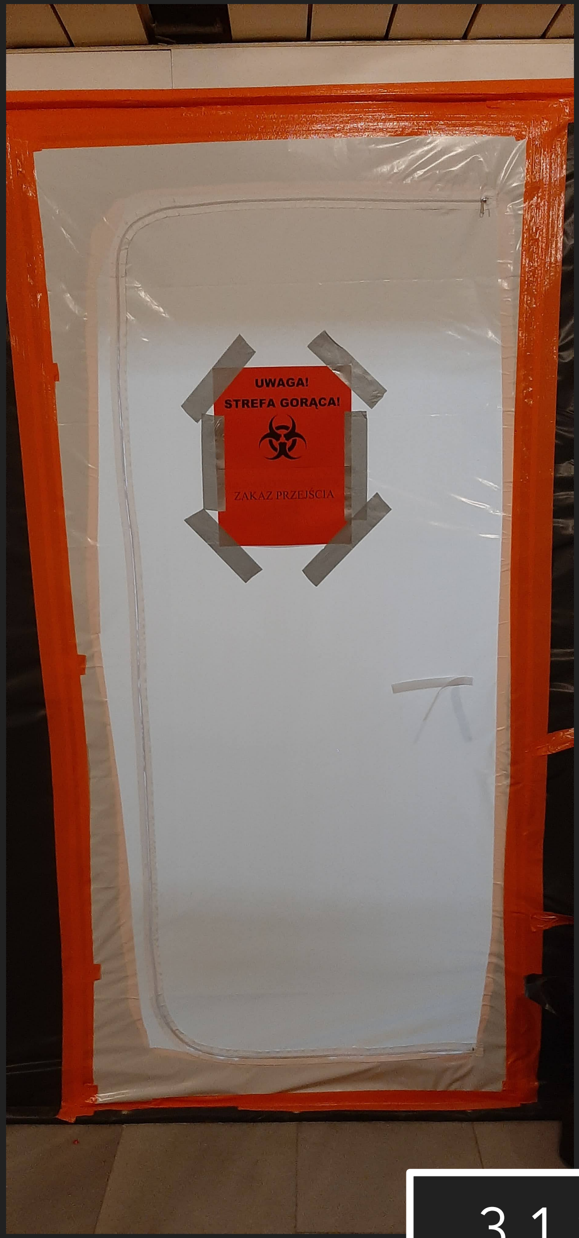

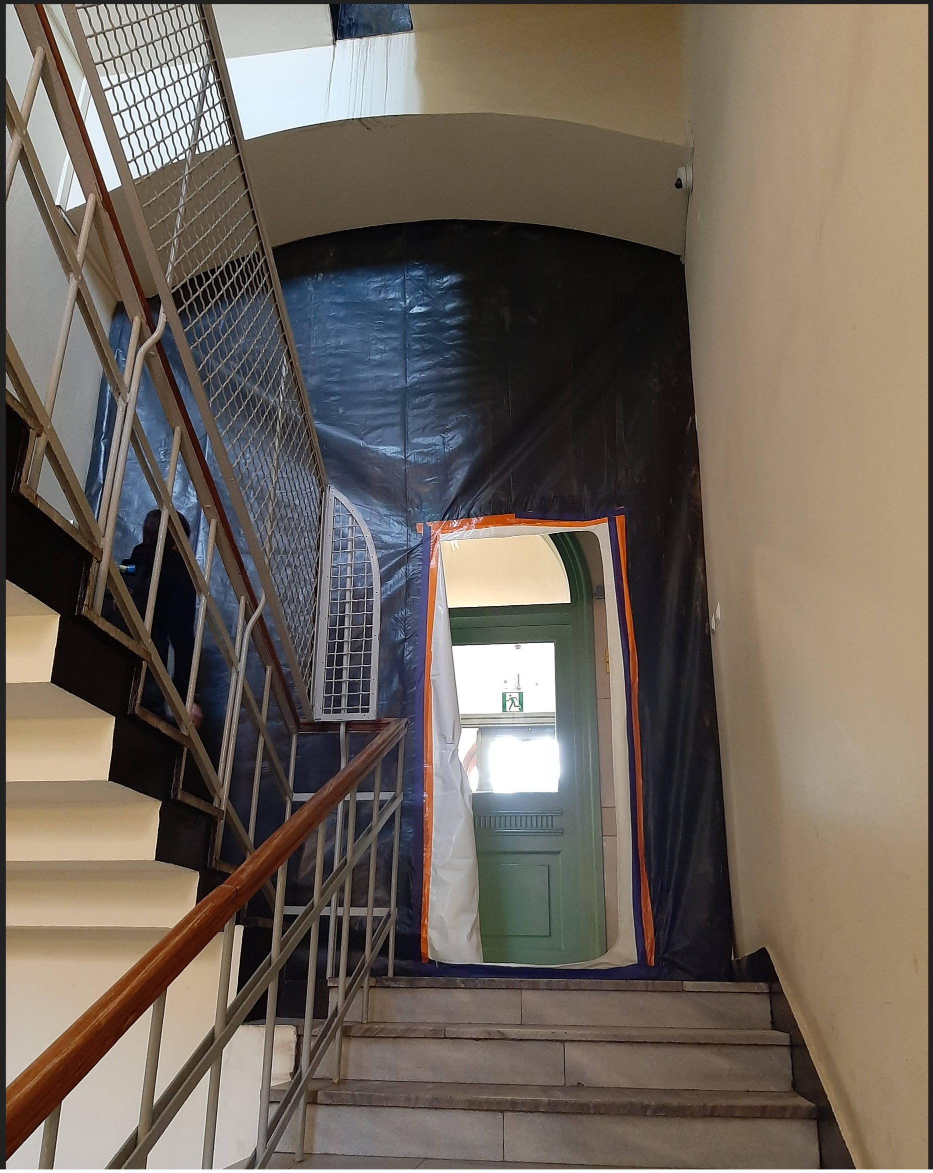

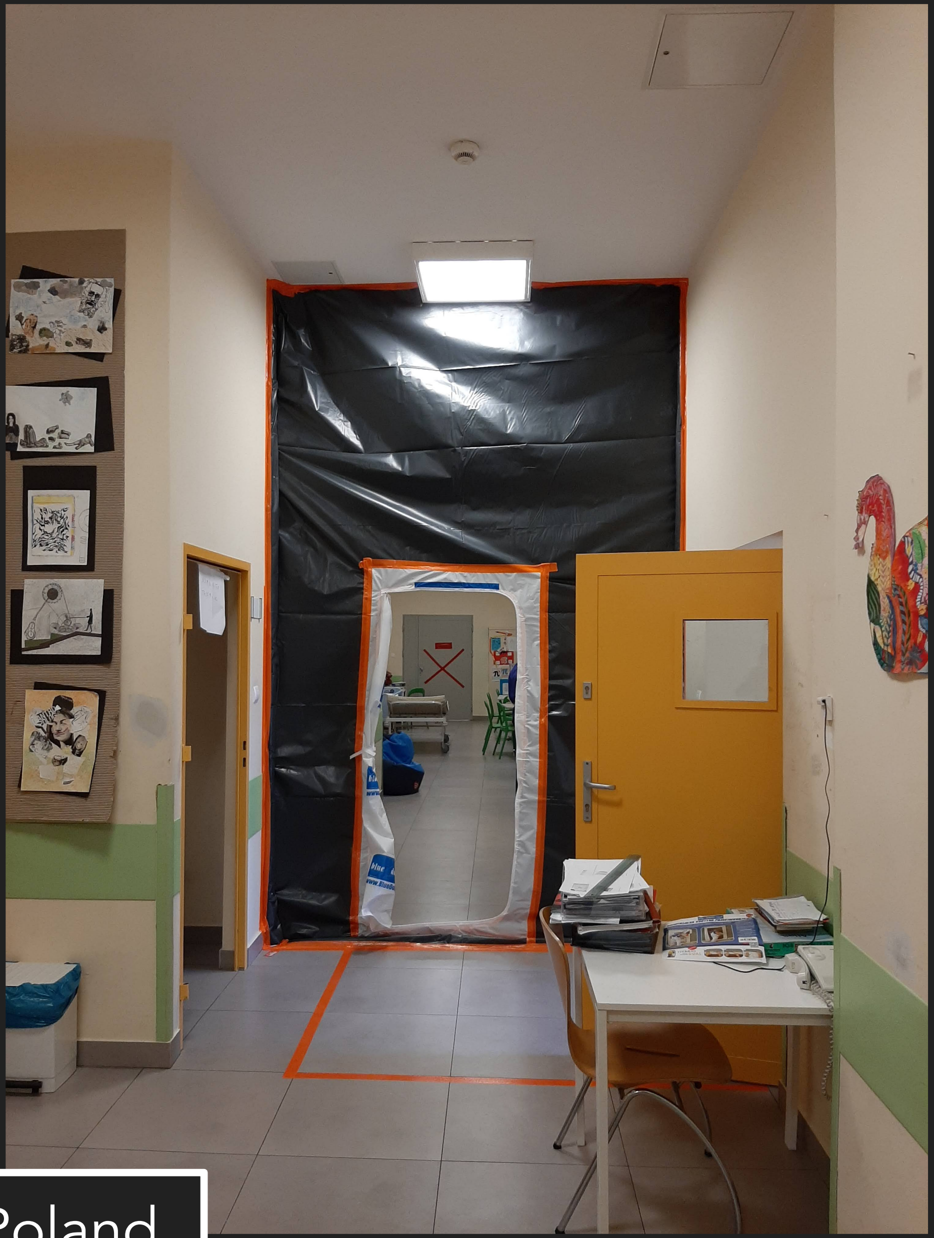

3.1 Service provision and delivery of care; Poland

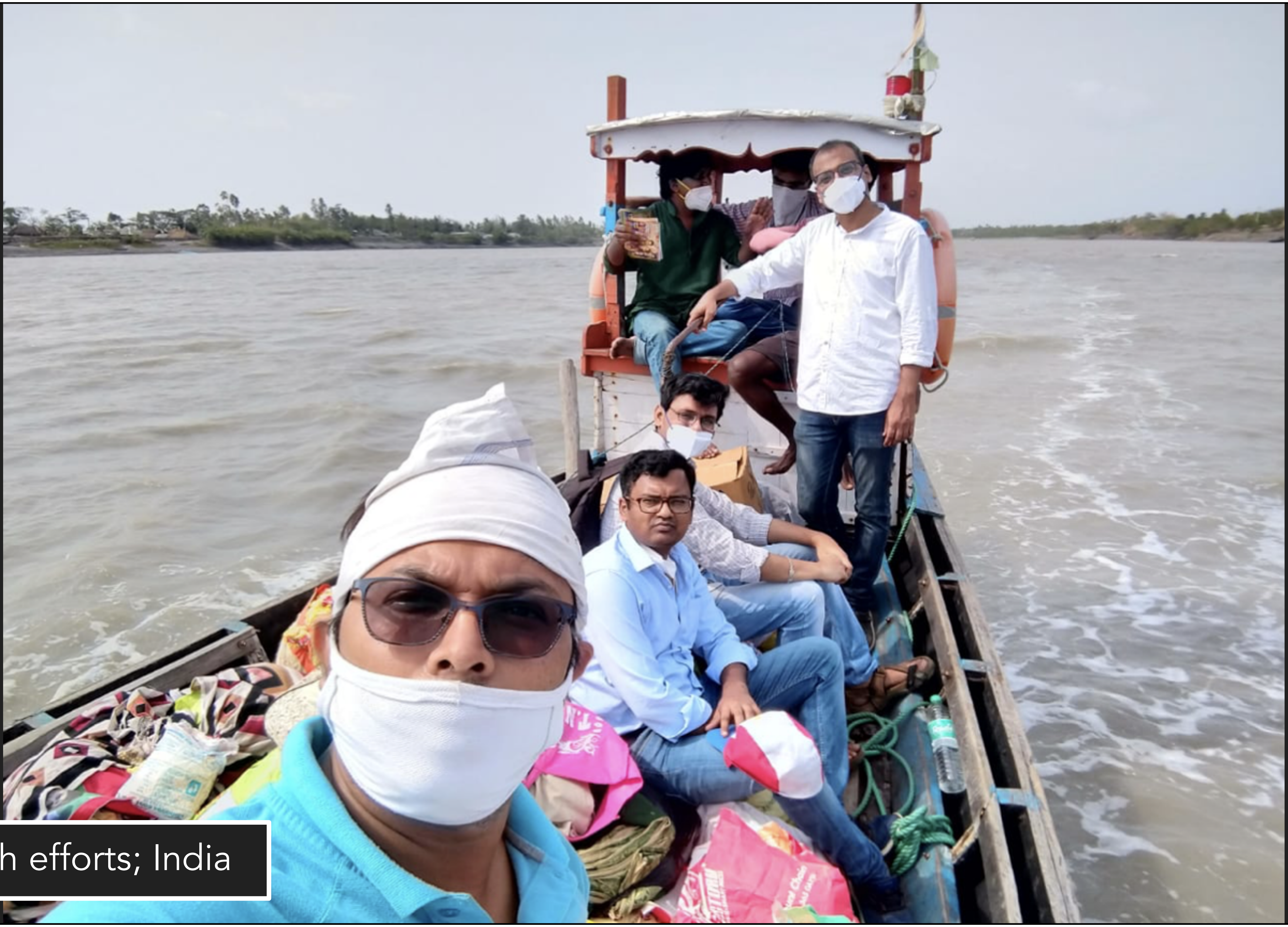

3.2 Outreach efforts; India

3.3 Guild pride; Cameroon

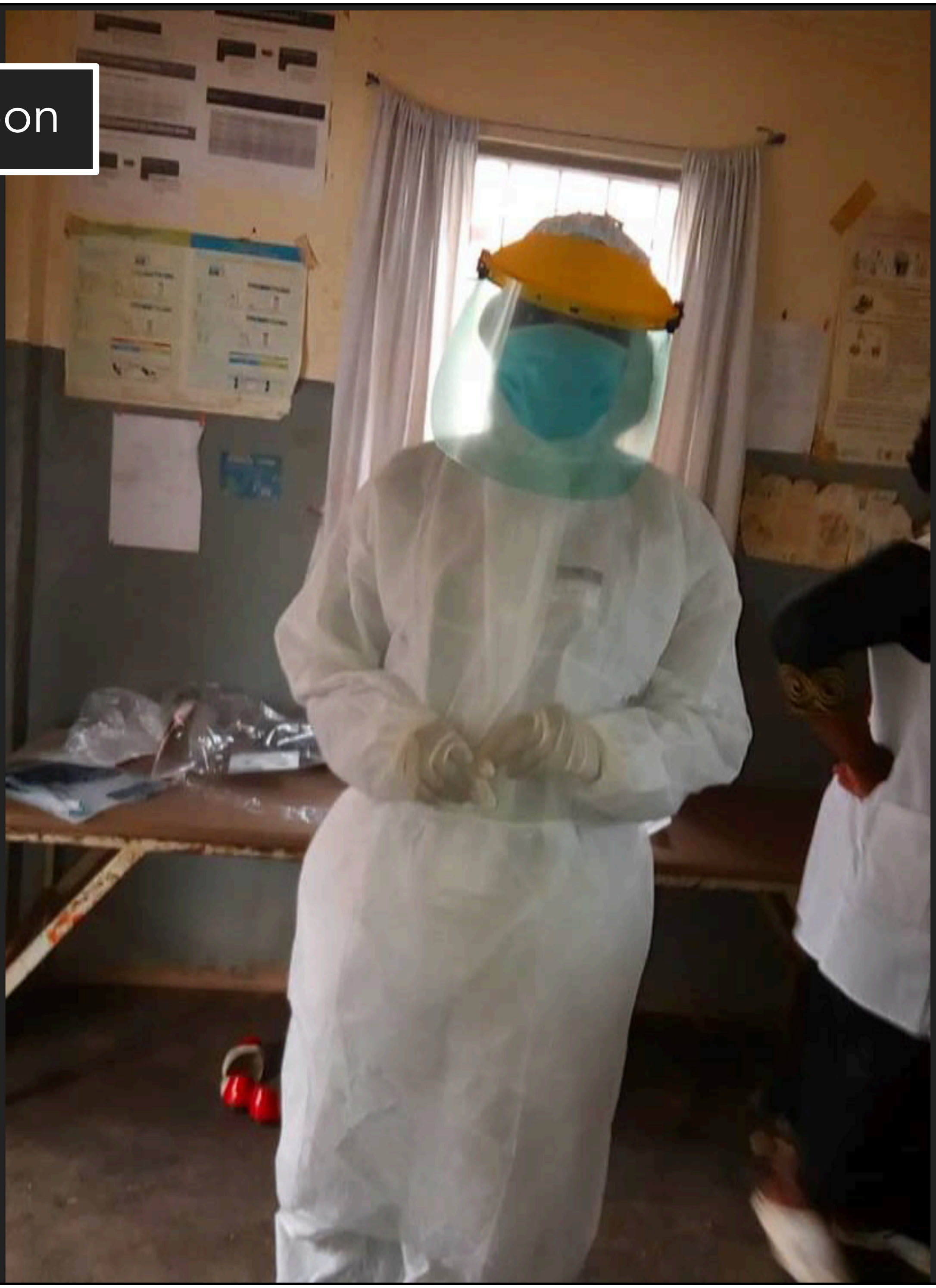

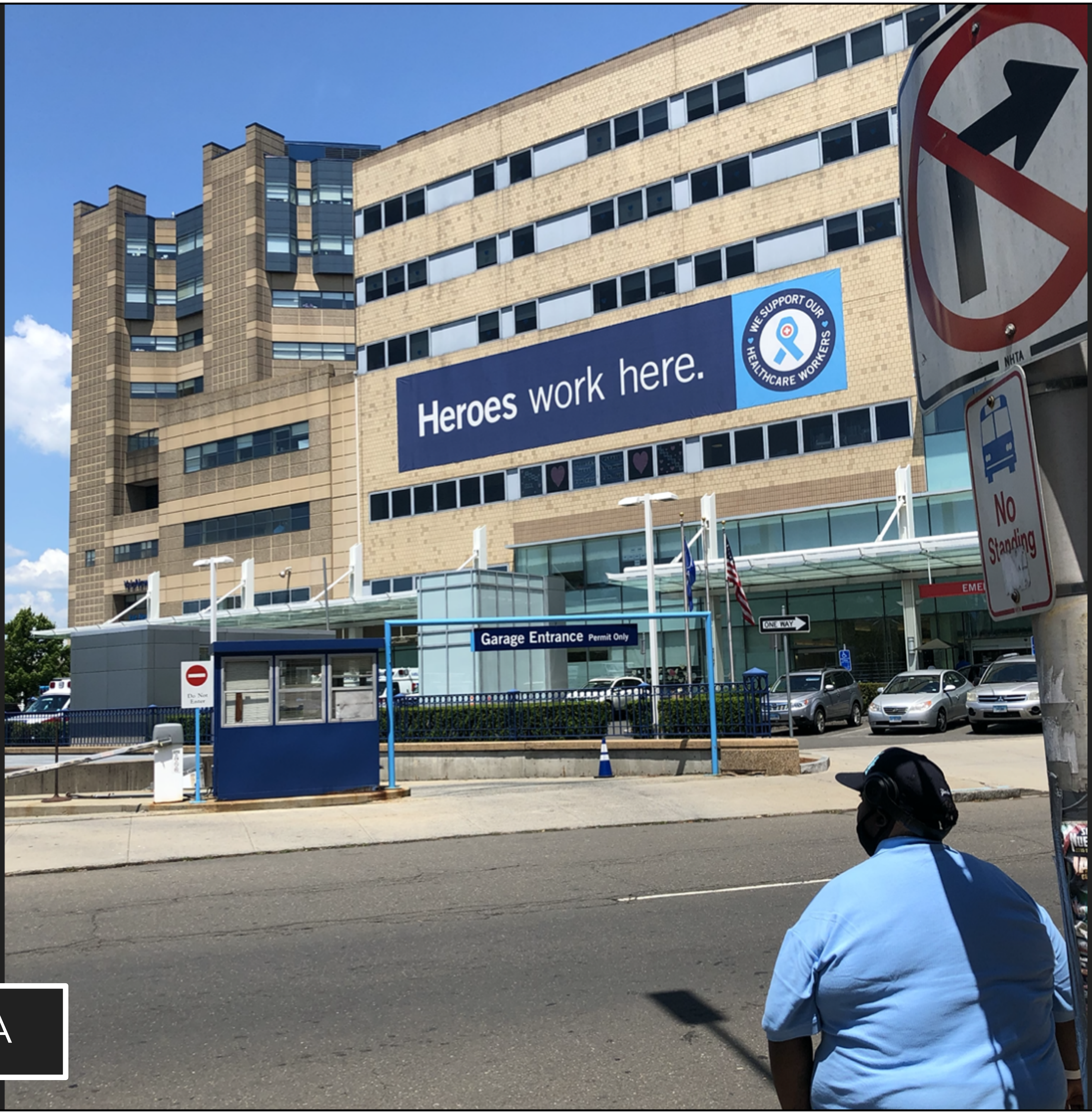

3.3 Guild guilt; USA

4. Purpose

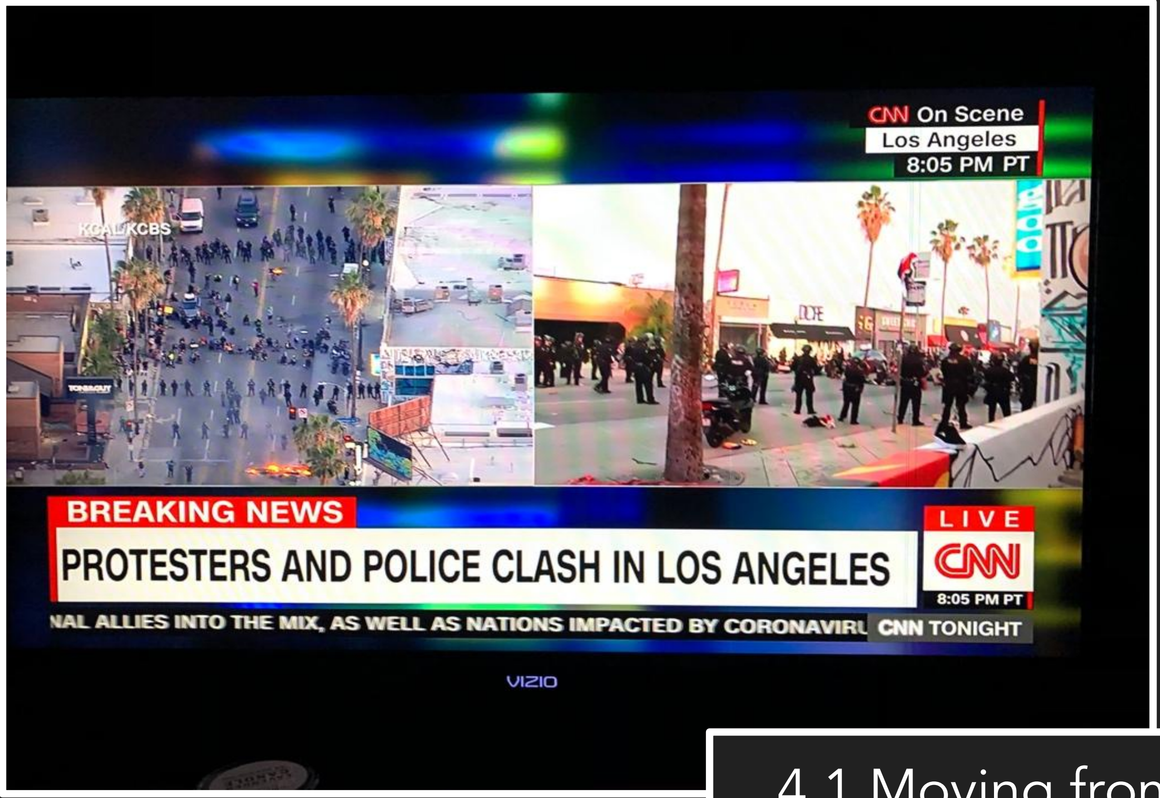

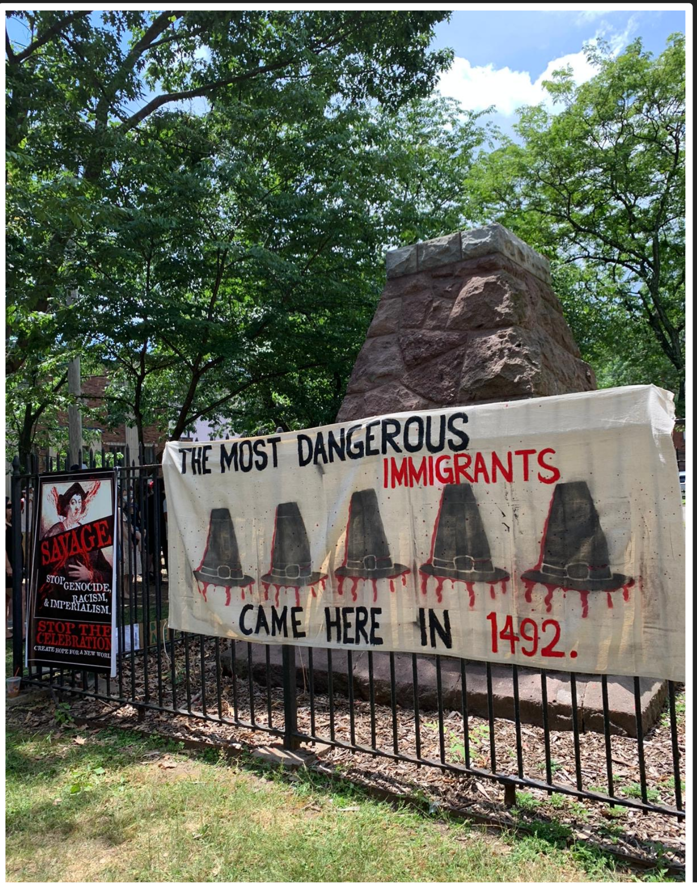

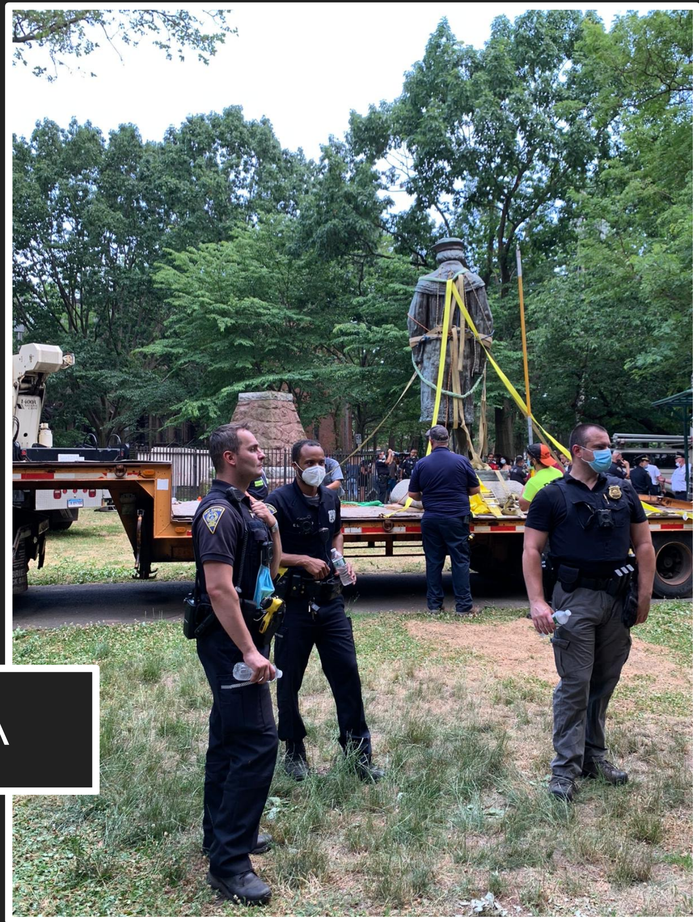

4.1 Moving from pandemic to syndemic; USA

4.2 Lamenting; South Africa

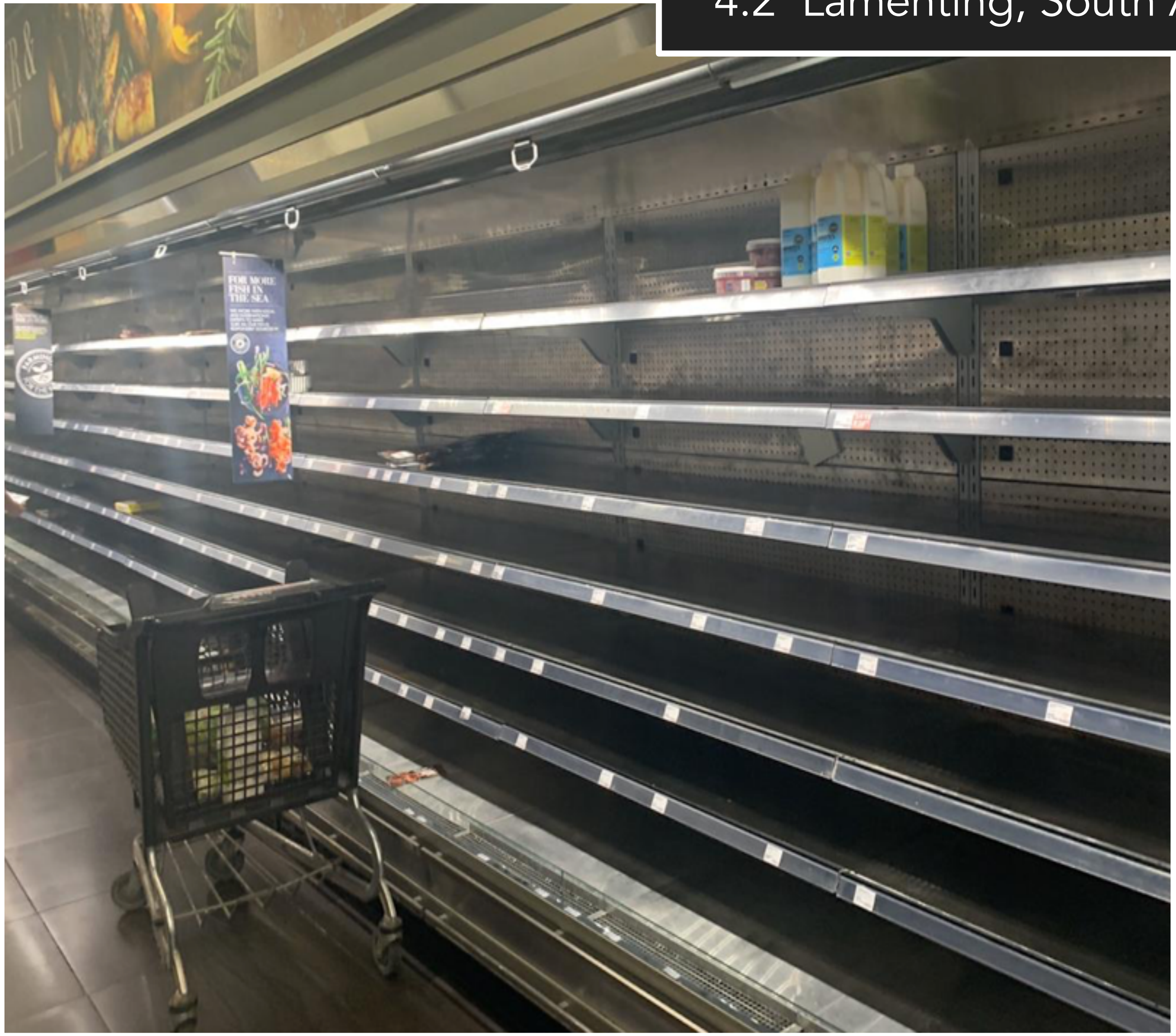

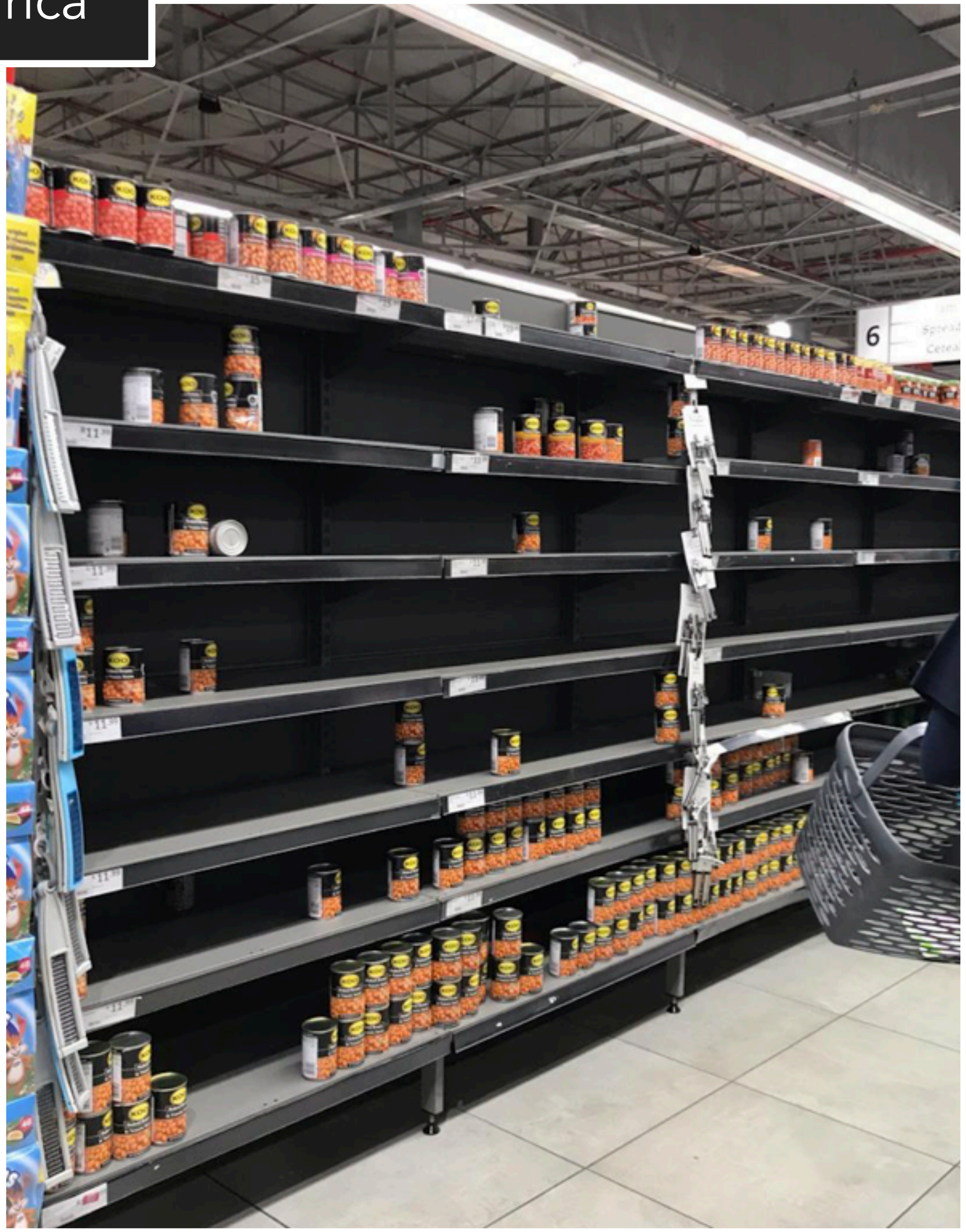

4.2 Embracing social responsibility; Chile

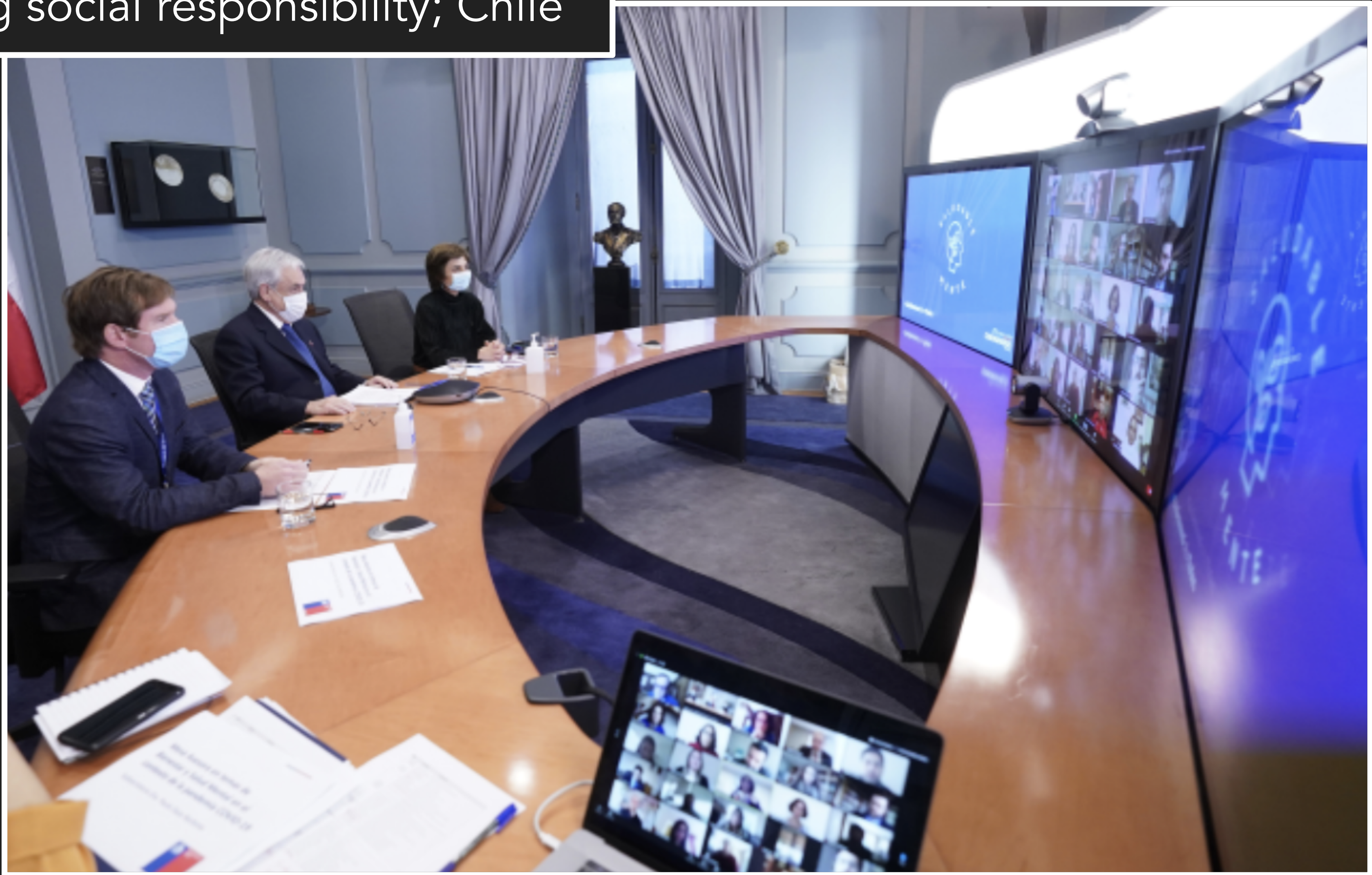

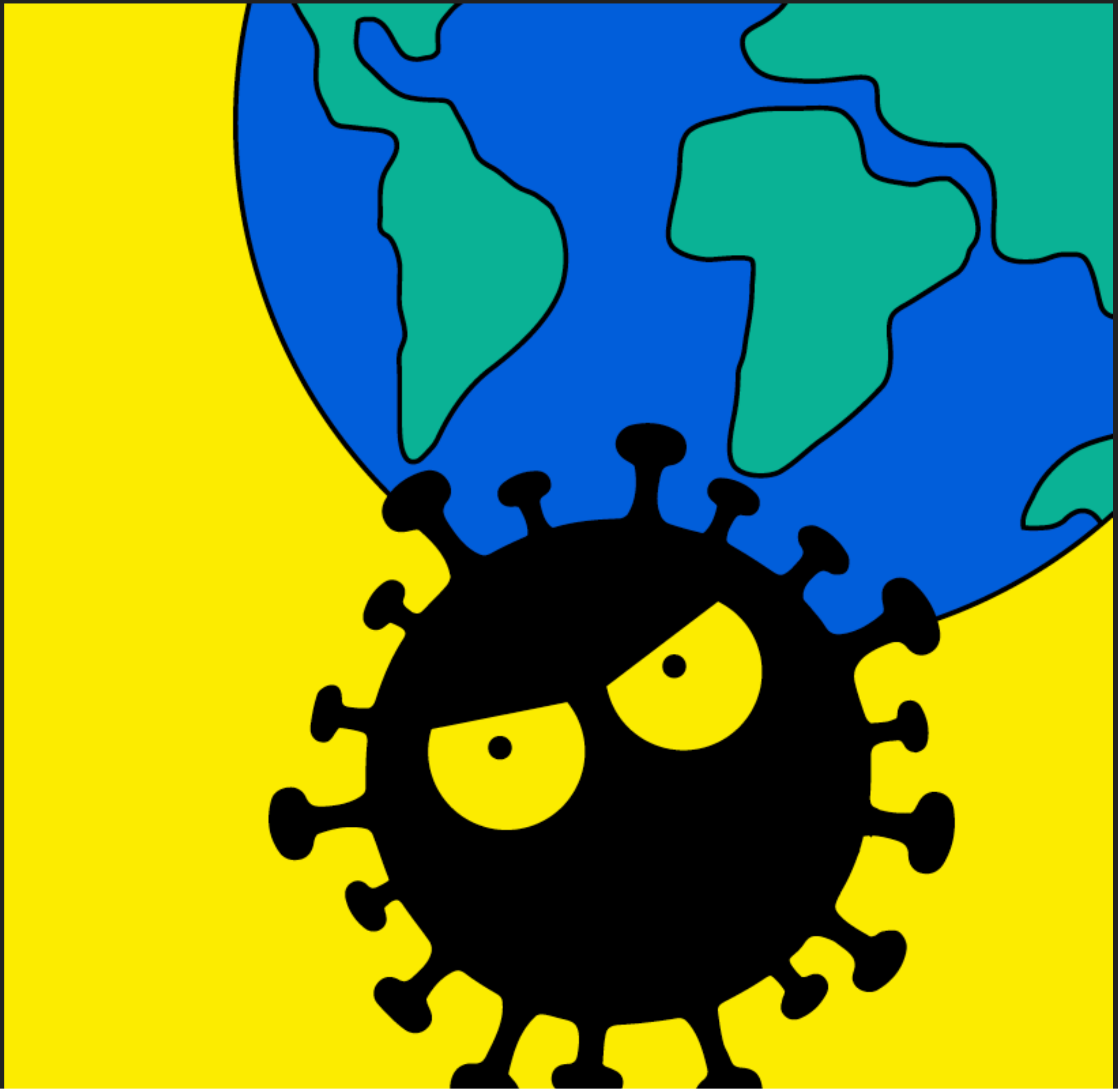

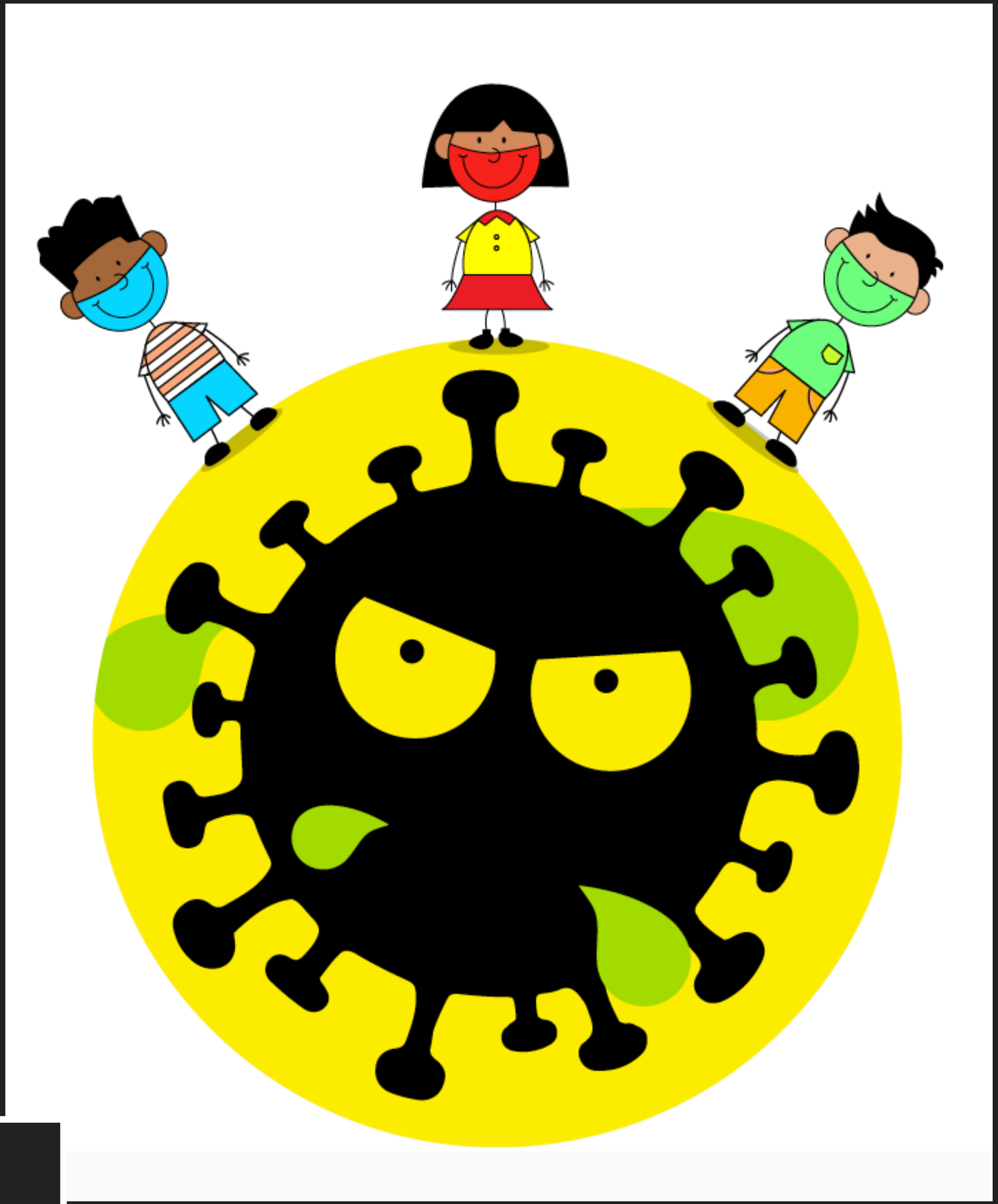

4.3 Planning toward a better tomorrow; South Africa
